# CT‐Visible Microspheres Enable Whole‐Body In Vivo Tracking of Injectable Tissue Engineering Scaffolds

**DOI:** 10.1002/adhm.202303588

**Published:** 2024-05-04

**Authors:** Annalisa Bettini, Peter Stephen Patrick, Richard M. Day, Daniel J. Stuckey

**Affiliations:** ^1^ Centre for Advanced Biomedical Imaging Division of Medicine University College London London WC1E 6DD UK; ^2^ Centre for Precision Healthcare Division of Medicine University College London London WC1E 6JF UK

**Keywords:** computed tomography, injectable scaffolds, microspheres, multimodal imaging, trackable

## Abstract

Targeted delivery and retention are essential requirements for implantable tissue‐engineered products. Non‐invasive imaging methods that can confirm location, retention, and biodistribution of transplanted cells attached to implanted tissue engineering scaffolds will be invaluable for the optimization and enhancement of regenerative therapies. To address this need, an injectable tissue engineering scaffold consisting of highly porous microspheres compatible with transplantation of cells is modified to contain the computed tomography (CT) contrast agent barium sulphate (BaSO_4_). The trackable microspheres show high x‐ray absorption, with contrast permitting whole‐body tracking. The microspheres are cellularized with GFP+ Luciferase+ mesenchymal stem cells and show in vitro biocompatibility. In vivo, cellularized BaSO_4_‐loaded microspheres are delivered into the hindlimb of mice where they remain viable for 14 days. Co‐registration of 3D‐bioluminescent imaging and µCT reconstructions enable the assessment of scaffold material and cell co‐localization. The trackable microspheres are also compatible with minimally‐invasive delivery by ultrasound‐guided transthoracic intramyocardial injections in rats. These findings suggest that BaSO_4_‐loaded microspheres can be used as a novel tool for optimizing delivery techniques and tracking persistence and distribution of implanted scaffold materials. Additionally, the microspheres can be cellularized and have the potential to be developed into an injectable tissue‐engineered combination product for cardiac regeneration.

## Graphical abstract

Highly porous microspheres containing barium sulfate are produced and then cellularized with bioluminescent mesenchymal stem cells to permit detection using X‐ray CT and optical imaging. Injectable microspheres are delivered in a minimally invasive manner under ultrasound guidance. This multi‐modal imaging approach allows co‐localization of cells and microspheres that can be studied longitudinally in vivo.

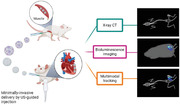

## 1 Introduction

Cell therapies have the potential to repair damaged organs including the heart. Numerous studies have attempted to transplant cells to regenerate the myocardium lost during myocardial infarction. However, to date, the preclinical benefits of cardiac cell therapy have only produced modest improvements in clinical trials, irrespective of the cell type used.^[^ ^1^, ^2^ ^]^

To ameliorate or treat chronic heart failure, cell therapies need to first be delivered to and disperse at the optimal location. Second, some cell therapies rely on integration with the host tissue as the presumed mechanism of action, where it is hoped that they will replace a substantial proportion of the myocardium lost to infarction. Although the therapeutic effect of cell therapy is premised on cell survival to benefit direct regeneration, paracrine signaling is also believed to play a regenerative role.^[^ ^3^, ^4^, ^5^, ^6^ ^]^ When direct replacement of the lost tissue is the goal, it is important that transplanted cells are retained in the heart for long enough to engraft and subsequently contribute to contractility.^[^ ^3^, ^4^, ^5^, ^6^ ^]^

Intravenous, intracoronary, and trans‐endocardial delivery of cell therapies in suspension is generally performed by minimally invasive catheter injections.^[^ ^6^ ^]^ However, myocardial cell retention via these delivery routes is low due to washout effects and cell death.^[^ ^6^ ^]^ Tissue scaffolds offer one possible solution to enhance cell retention, providing a substrate for cell adhesion and the creation of de novo tissue.^[^ ^7^, ^8^, ^9^, ^10^, ^11^ ^]^ Tissue scaffolds can be surgically implanted as patches, or injected as viscous hydrogels (see reviews by Tenreiro et al.^[^ ^12^ ^]^ and Montero et al.,^[^ ^13^ ^]^) though particle‐based scaffold systems are also emerging.

Cardiac patches and engineered heart tissues (EHTs) comprise of cells attached to a scaffold which encourages cell survival and facilitates functional cell‐cell contact, resulting in a structured cellularized scaffold construct.^[^ ^14^ ^]^ To implant cardiac patches, epicardial attachment is normally performed via direct surgical access to the heart through a thoracotomy.^[^ ^15^, ^16^, ^17^, ^18^, ^19^ ^]^ However, invasive open‐chest surgery increases the risk of intervention‐related morbidities and limits the clinical application.^[^ ^6^ ^]^ Although cardiac patches and EHTs have been shown to improve cell retention and cardiac function,^[^ ^14^, ^20^ ^]^ they are frequently isolated from the host myocardium by reactive epicardial fibrosis which can prevent effective contractile and electrophysiological synchrony.^[^ ^16^, ^21^ ^]^

Injectable cell encapsulating hydrogels have been generated from a range of synthetic and natural materials including, alginate, polyethylene glycol (PEG), chitosan and poly(lactide‐co‐glycolide) PLGA.^[^ ^8^, ^22^, ^23^ ^]^ Advantages of hydrogels include simple mixing with cells and biologics just prior to injection and minimally invasive delivery as a liquid via fine gauge needles before crosslinking in situ. Gelation and degradation time can be modified by the choice of material and cross‐linking mechanism.^[^ ^8^, ^22^, ^23^ ^]^ Injectable hydrogels, are clinically desirable as they negate the need for surgical implantation, but cell‐cell contact and structure are often missing resulting in lower cells retention than implanted 3D constructs.^[^ ^16^ ^]^

Here, we have utilized an injectable microsphere scaffold that can foster cell attachment and cell‐cell contact, combining the benefits of both hydrogel and cardiac patches. This approach allows minimally invasive cell delivery and dispersal of the tissue engineered construct throughout the myocardium to encourage integration and better cell survival.

The ability to track transplanted cells and scaffold materials is of paramount importance for the optimization of material formulation, mode of administration, and delivery route, ultimately maximizing regeneration at the target site and minimizing off‐target effects.^[^ ^24^, ^25^, ^26^, ^27^ ^]^ Importantly, a method to visualize the location of grafted cells and scaffolds after delivery could be used to optimize therapies by verifying injection success, retention of the transplanted cells, and distribution of the product.

Several methods have been developed for tracking the location of injected cells using medical imaging. These include direct labeling with iron oxides for MRI^[^ ^28^ ^]^ or radionuclides for PET and SPECT.^[^ ^29^ ^]^ However, direct cell labeling has several disadvantages including lack of specificity, as the label can be lost from the donor cell, passed to host tissues and continue to give signal even in dead cells, and toxicity due to the label being internalized within the transplanted cells.

Tracking of tissue engineering scaffolds can overcome these problems and be used to give real time in vivo data on location, migration, and degradation. A trackable scaffold can potentially be used as an off‐the‐shelf product for any compatible cell therapy. Scaffold labeling can be used in tandem with imaging of luciferase reporter genes for cell tracking, which although not clinically translatable, can provide a sensitive method for tracking the location and viability of donor cells in pre‐clinical models.^[^ ^30^, ^31^ ^]^ This multi‐modal imaging approach could for the first time allow the co‐localization of cells and tissue engineering scaffolds to be studied longitudinally in vivo. The combination of novel delivery strategies and accurate tracking approaches will be beneficial in assessing the impact of tissue engineering approaches for cell retention in the heart.

Here, we have developed an injectable cell‐substrate consisting of highly porous implantable microspheres containing the computed tomography (CT) contrast agent barium sulphate (BaSO_4_). This approach enabled minimally invasive delivery of cells attached to the microsphere substrate and whole‐body in vivo tracking of the injected scaffold to determine the accuracy of delivery.

## 2 Results

### 2.1 X‐Ray Attenuation by BaSO_4_‐Loaded Microspheres

**Figure 1:**
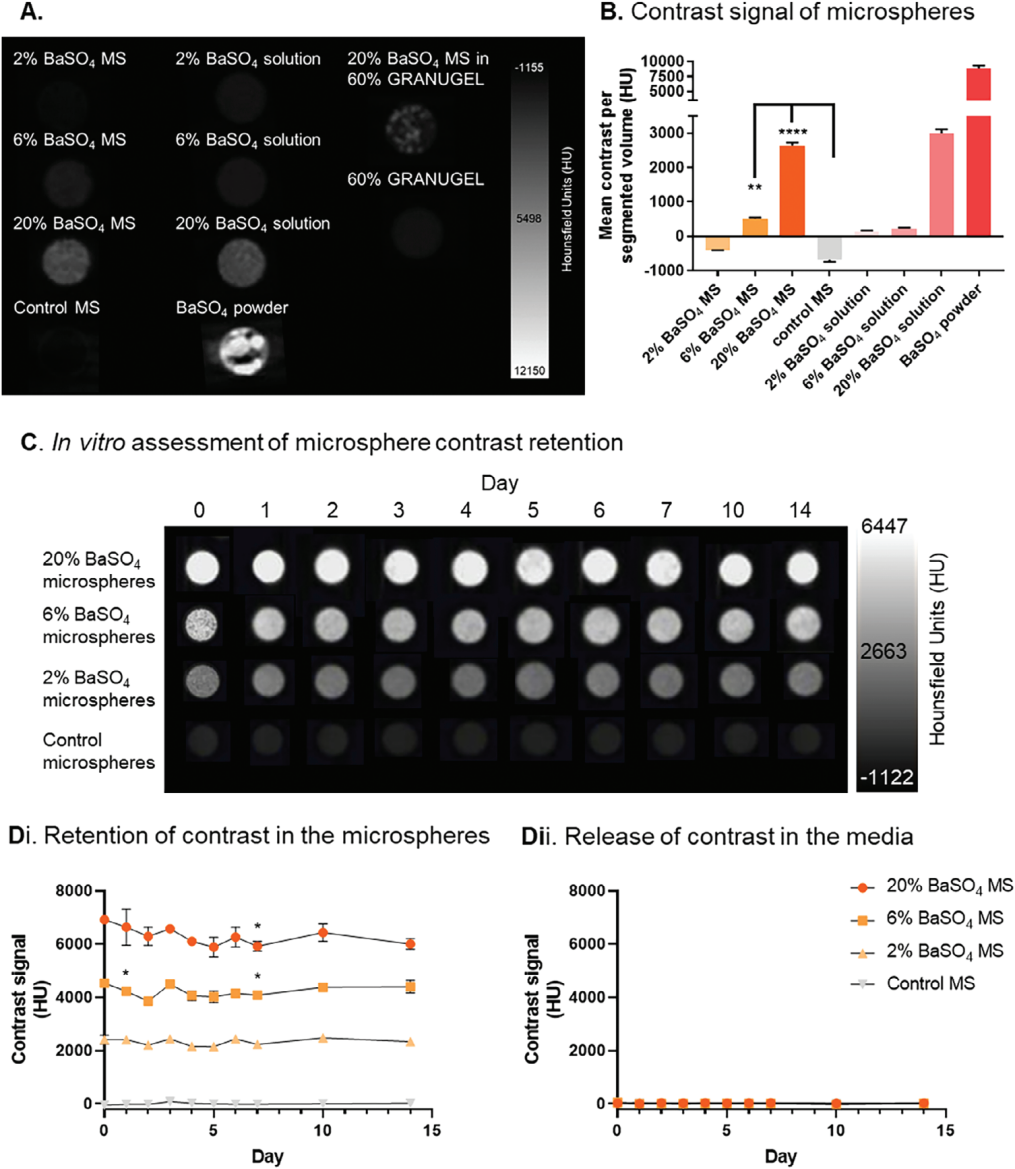
Fabrication of BaSO_4_‐loaded microspheres. A) µCT cross‐section and B) quantification of the contrast produced by BaSO_4_‐loaded microspheres, control microspheres, BaSO_4_ solution and Granugel dilution for suspension delivery. The contrast signal was retained in BaSO_4_‐loaded microsphere formulations up to 14 days in vitro. C) µCT cross‐section and D) quantification of the contrast (i) produced and released (ii) by BaSO_4_‐loaded microspheres in vitro. Data are presented as mean ±SD. The significance of the data was calculated by (B) One‐way ANOVA with Tukey's post‐hoc analysis and (D) Two‐way ANOVA with Tukey's post‐hoc analysis. (n = 3, **P = 0.0054, ****P<0.0001). Microspheres (MS).

Microspheres were loaded with CT contrast agent BaSO_4_ during the fabrication process and were compared with control microspheres without BaSO_4_ (**Figure** **1**; Figure S1, Supporting Information). 2 – 20% (w/v) BaSO_4_‐loaded microspheres were produced and their radiopacity was measured by µCT. 2 – 6% BaSO_4_‐loaded microspheres produced mean contrast levels higher than control microspheres, but below the contrast level of soft tissue (Figure 1A,B). The loading of BaSO_4_ was increased to obtain a contrast measurement above the level of soft tissue and bone. 20% BaSO_4_‐loaded microspheres produced contrast above 2000 HU (Figure 1A,B). This value was above the attenuation factors of soft tissue and bone, which suggested that the microspheres would be distinguishable for tracking in vivo.

In vitro release studies confirmed that the BaSO_4_‐loaded microspheres retained contrast up to 14 days, with signal attenuation remaining constant over this period (Figure 1C,D(i)). Collection of spent media also confirmed that contrast was not released from the microspheres at a detectable level, confirming the stability of the BaSO_4_‐loaded microspheres (Figure 1Dii).

### 2.2 Characterization of BaSO_4_‐Loaded Microspheres

**Figure 2:**
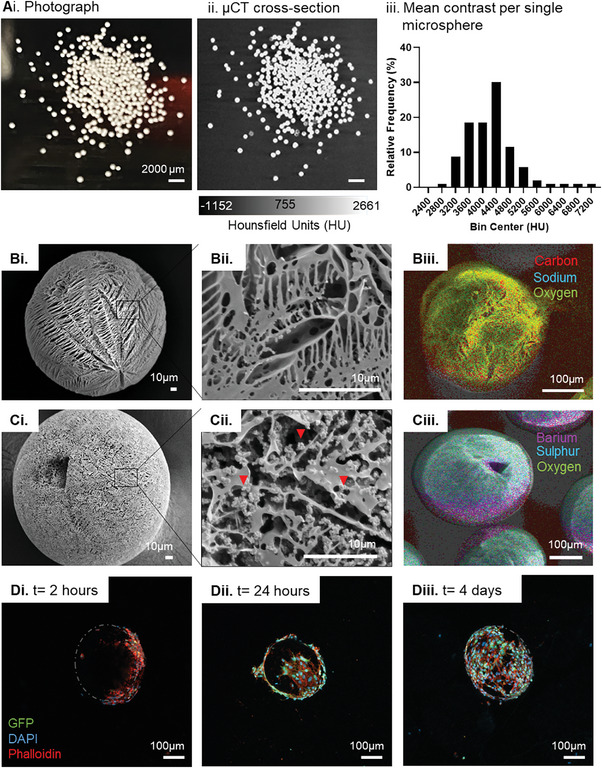
In vitro characterization of 20% BaSO_4_‐loaded microspheres. A) Visibility of single microspheres (20% BaSO_4_‐loaded) confirmed by light microscopy and µCT. Physical characterization of a single B) control microsphere without BaSO_4_ and C) 20% BaSO_4_‐loaded microsphere by (i‐ii) SEM, red arrows indicate the presence of BaSO_4_ crystals, and (iii) EDX analysis. D) Confocal micrographs of MSC attached to a 20% BaSO_4_‐loaded microsphere at (i) 2‐hours and (ii) 24‐hours and (iii) 4‐days post‐seeding. Grey dotted line outlines a single microsphere. Data are presented as relative frequency of mean contrast per segmented microsphere, n = 104.

To determine whether individual 20% BaSO_4_‐loaded microspheres were trackable, light micrographs and µCT cross sections of 20% BaSO_4_‐loaded microspheres were compared (**Figure** **2**A). The contrast generated by the microspheres in the µCT cross sections (Figure 2Ai) matched the visual outline of the microspheres in the light micrographs (Figure 2Aii), confirming that individual 20% BaSO_4_‐loaded microspheres were detectable. The variability of contrast generated by single microspheres was also quantified in 104 microspheres (Figure 2Aiii). Contrast ranged between 2800–7200 HU and median contrast produced by a single microsphere was 4400 HU.

The morphology of the 20% BaSO_4_‐loaded microspheres was assessed using SEM. Loading of BaSO_4_ resulted in the porosity of the microspheres being retained but led to a change in the surface topography. Surface pores were visible with a regular pore pattern, but the pores were smaller in size (Figure 2B,C). The loading of BaSO_4_ was visible on the surface of the microsphere as evident at higher magnifications (Figure 2Cii).

BaSO_4_‐loaded microspheres were further characterized using Energy Dispersive X‐Ray Spectroscopy (EDX) and compared to control microspheres without BaSO_4_ (Figure 2Bii,Cii; Figure S1, Supporting Information). Representative electron images and elemental analysis of control microspheres indicated the presence of carbon and oxygen on the surface of the control microspheres (Figure 2Biii). EDX analysis in Figure 2Ciii confirmed the presence of barium and sulfur in the BaSO_4_‐loaded microspheres.

### 2.3 Culturing Cells on BaSO_4_‐Loaded Microspheres

The 20% BaSO_4_‐loaded microspheres were carried forward for further in vitro and in vivo characterization. The microspheres were seeded with murine MSCs, a cell type chosen due to their therapeutic potential and species compatibility.

The attachment of murine MSC to 20% BaSO_4_‐loaded microspheres was monitored by light microscopy and expansion was confirmed by confocal microscopy (Figure 2D). MSC were seeded onto microspheres and became attached to the microspheres within 2 hours post‐seeding (Figure 2Di). In 24 hours, cell coverage expanded over the surface of the microspheres (Figure 2Dii). After 4 days, MSC showed integration and total coverage of the surface of the microspheres. Expansion of the cell coverage on the surface of the microspheres was due to cell proliferation. The significant increase in total MSC cell count at 24‐ and 48‐ hours post‐seeding suggests the occurrence of cell proliferation and was further confirmed by fluorescent microscopy (Figures S2A and S3, Supporting Information).

**Figure 3:**
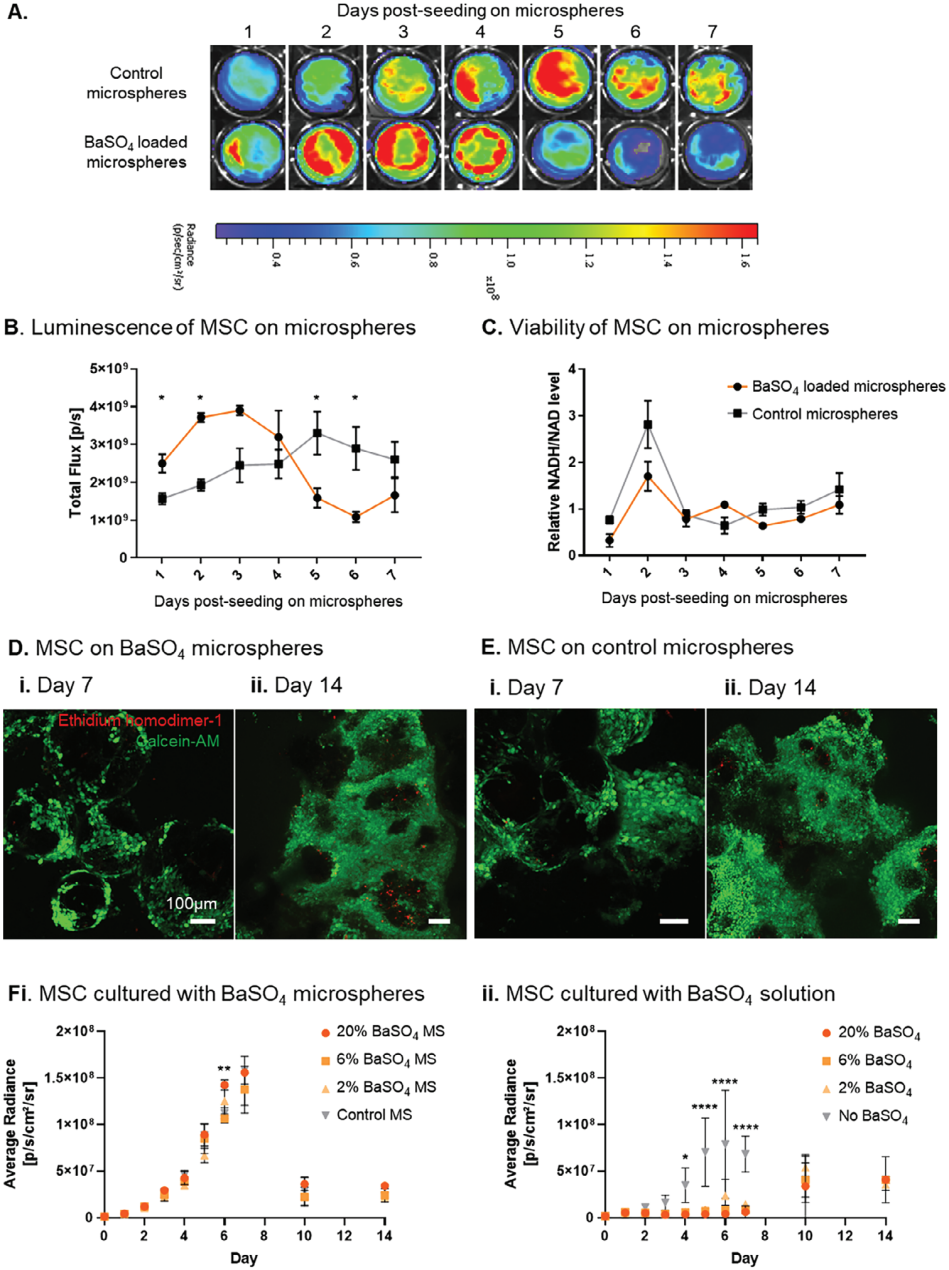
In vitro characterization of MSC viability on microspheres. A) Luminescence of MSC on 20% BaSO_4_ loaded‐ and control‐ microspheres. B) Quantification of luminescence activity and C) metabolic activity of MSC on microspheres over 7 days. Live/Dead stained confocal micrographs of MSC on D) 20% BaSO_4_ loaded‐ and E) control‐ microspheres at 7 and 14 days post seeding. F) Assessment of direct BaSO_4_ cytotoxicity to MSC culture. MSC luminescence after exposure to (i) BaSO_4_ loaded microspheres and (ii) matched concentrations of BaSO_4_ solutions. Data are presented as mean ±SD. The significance of the data was calculated by (B,C) Unpaired t‐test with Holm Sidak's post‐hoc analysis and (F) Two‐way ANOVA with Dunnett's post‐hoc analysis, (n = 3, *P<0.05, **P<0.01, ****P<0.0001). Microspheres (MS).

The viability of MSC attached to 20% BaSO_4_‐loaded microspheres was assessed by luminescence, XTT assays and live/dead staining (**Figure** **3**).

Due to the ATP dependence of luciferase signal, luminescence reflects the number of viable, metabolically active cells.^[^ ^32^ ^]^ Luminescent activity correlated with counts of MSC seeded at 8.0 × 10^5^ through 8.0 × 10^2^ (Figure S2, Supporting Information). The luminescent signal of MSC on BaSO_4_‐loaded microspheres peaked at day 3 post‐seeding (3.9× 10^9^ flux), and on control microspheres peaked at day 5 post‐seeding (3.3× 10^9^ flux) (Figure 3A,B). These differences in cell growth kinetics resulted in luminescent signal being significantly different between the two groups at days 1, 2, 5, and 6 post‐seeding.

NADH and NADPH metabolism was measured by XTT assay, giving comparable results for control and BaSO_4_‐loaded microspheres (Figure 3C). Peak metabolic activity was measured on day 2 post‐seeding followed by sustained metabolic activity up to day 7 post‐seeding.

Live/ Dead staining confirmed the viability of MSC on both control and 20% BaSO_4_‐loaded microspheres up to 14 days (Figures S3 and S4, Supporting Information). Majority of MSC stained positive for Calcein‐AM at all time points, while positive dead cell staining was minor and comparable to the control sample (Figure 3D). The extended time course is included in Figures S3 and S4 (Supporting Information).

To further confirm in vitro biocompatibility, the direct cytotoxicity of BaSO_4_ on MSC culture was assessed by comparing the luminescence of MSC in transwell culture with either BaSO_4_‐loaded microspheres or matching concentrations of BaSO_4_ solutions (Figure 3F). As expected, MSC cultured with BaSO_4_ solutions displayed significantly lower luminescence compared to the control samples cultured without BaSO_4_ (Figure 3Fii). Light micrographs confirmed poor cell survival and expansion following exposure to BaSO_4_ solutions (Figure S5, Supporting Information). In contrast, the luminescence of MSC cultured with BaSO_4_‐loaded and control microspheres, containing no BaSO_4_, was comparable. Luminescent signal increased up to day 7 due to cell expansion and followed typical cell proliferation dynamics (Figure 3Fi; Figure S6, Supporting Information).

Overall, the results suggested 20% BaSO_4_‐loaded microspheres were biocompatible with MSC culture and the formulation was taken forward for in vivo trackability studies.

### 2.4 In Vivo Tracking of Cell Loaded BaSO_4_‐Loaded Microspheres in Mouse Hindlimbs

**Figure 4:**
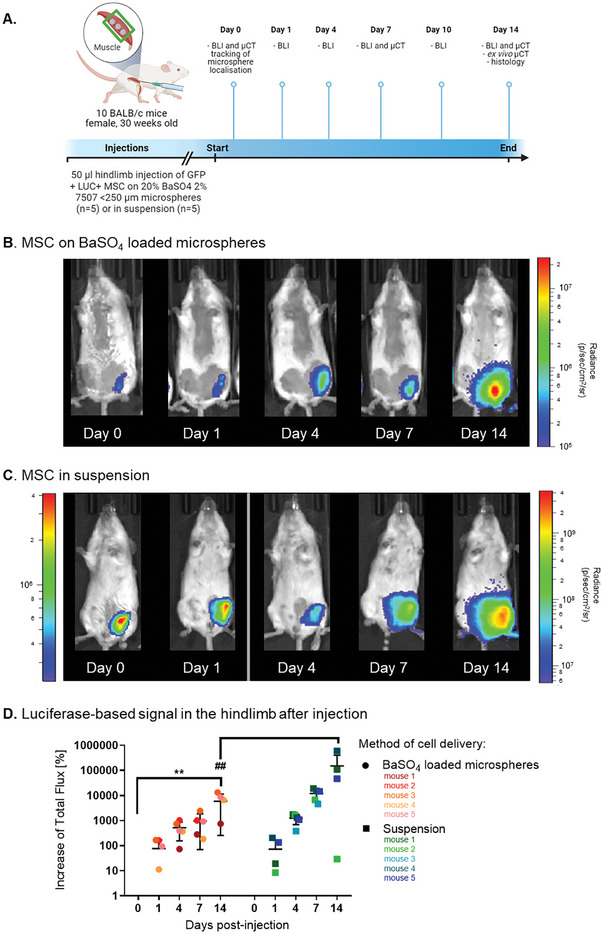
In vivo tracking and retention of MSC luminescence by BLI. A) Diagrammatic representation of the delivery protocol for MSC cellularized BaSO_4_‐loaded microspheres into a mouse hindlimb model. Time course of BLI images of luciferase positive MSC, B) delivered on BaSO_4_‐loaded microspheres or C) suspension, in the hindlimb. D) Quantification of the luminescent signal in the hindlimb over 14 days. Data are presented as mean ±SD. The significance of the data was calculated by One‐way ANOVA with Dunnett's post‐hoc analysis, (n = 5, **P = 0.0027, ##P = 0.0063).

BALB/c mice (n = 10) received hindlimb injections of 50 µL of 20% BaSO_4_‐loaded microspheres seeded with murine MSC or the same number of MSC in suspension (**Figure** **4**A).

To monitor cell viability, proliferation and retention at low‐resolution, BLI was performed on days 0, 1, 4, 7, and 14 post‐injection (Figure 4B–D). The luciferase‐based signal of MSC delivered on microspheres significantly increased over the course of the 14 days, with maximum signal of 8.42 × 10^7^ flux (±7.29×10^7^ SD, **P = 0.0027, One‐way ANOVA with Dunnett's post‐hoc analysis, n = 5). The luciferase signal of MSC delivered in suspension also significantly increased (1916.8 × 10^7^ flux ±2744.1 × 10^7^ SD, ##P = 0.0063, One‐way ANOVA with Dunnett's post‐hoc analysis, n = 5).

**Figure 5:**
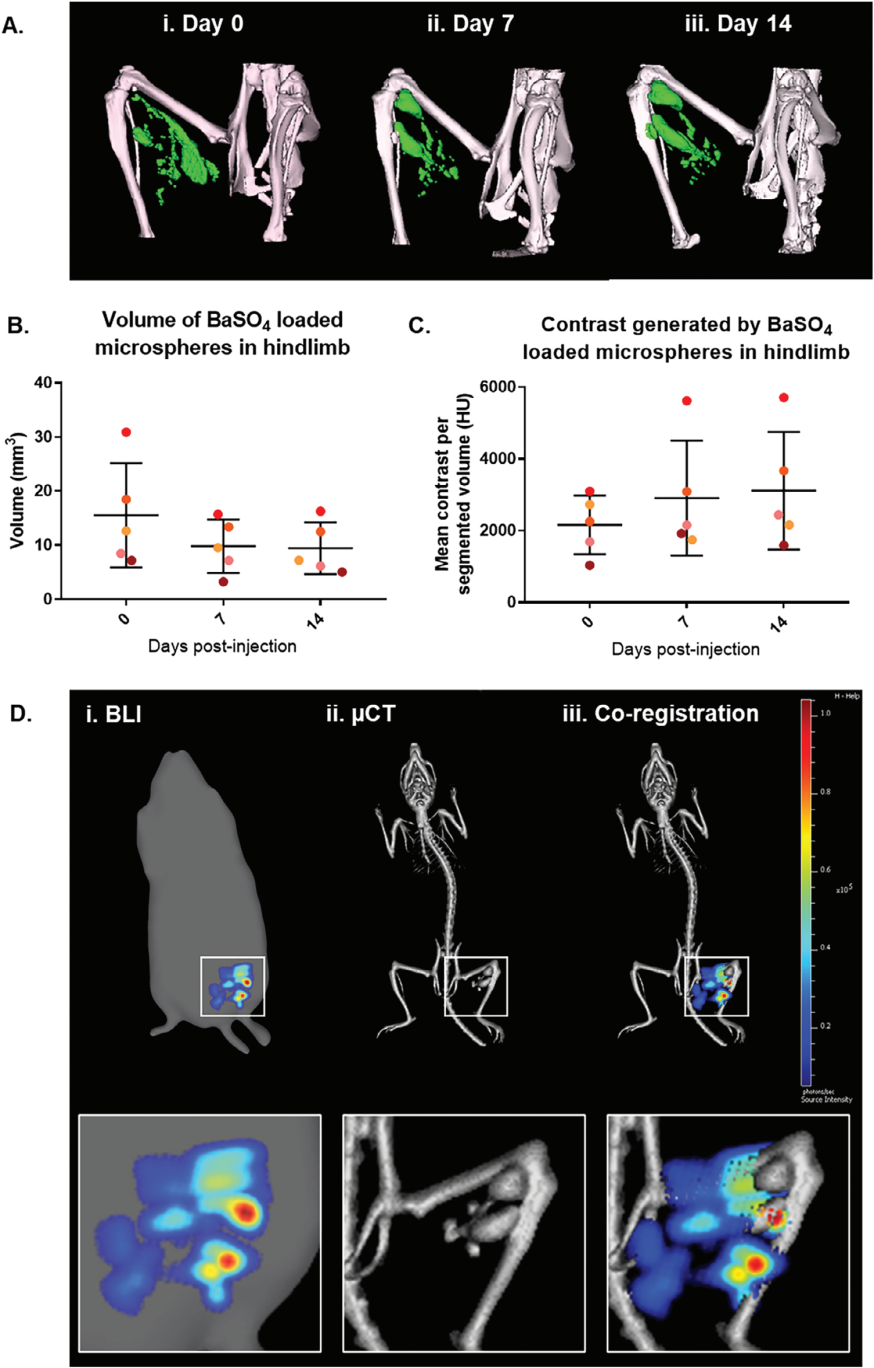
In vivo tracking and retention of the microspheres in the hindlimb as shown by µCT. A) Volume‐rendered 3D reconstructions of the contrast produced by BaSO_4_ microspheres on days 0, 7, and 14. Quantification of the B) total volume and C) contrast produced by BaSO_4_ microspheres in the hindlimb. D) Independent tracking of cell location by (i) BLI, and scaffold location by (ii) µCT. (iii) Co‐registering the 3D volume rendered BLI and µCT reconstructions enables assessment of the scaffold and cell co‐localization. Data are presented as mean ±SD. The significance of the data was calculated by One‐way ANOVA with Tukey's post‐hoc analysis. (n = 5, P>0.05). (Hounsfield Units – HU).

To monitor microspheres retention and biodistribution at high‐resolution, µCT was used to track the microspheres in the hindlimb and across the whole body (**Figure** **5**). Representative 3D volume rendered reconstructions for all animals are available in Figure S7 (Supporting Information). Quantification of the 3D volume rendered reconstructions showed that the total volume and contrast signal generated by the BaSO_4_‐loaded microspheres were similar from day 0 to 14 (Figure 5B,C).

3D BLI data was acquired and co‐registered with whole body µCT to allow for the first time the co‐localization of grafted cells and their scaffold substrate to be studied longitudinally in vivo (Figure 5D; Figure S8, Supporting Information). Signals remained co‐localized throughout the 14 days as would be expected for hindlimb injections of MSCs, but this approach could be valuable when delivering cells to target sites where cell retention is difficult, such as the heart.

### 2.5 In Vivo Tracking of BaSO_4_‐Loaded Microspheres after Ultrasound Guided Intramyocardial Injection

**Figure 6:**
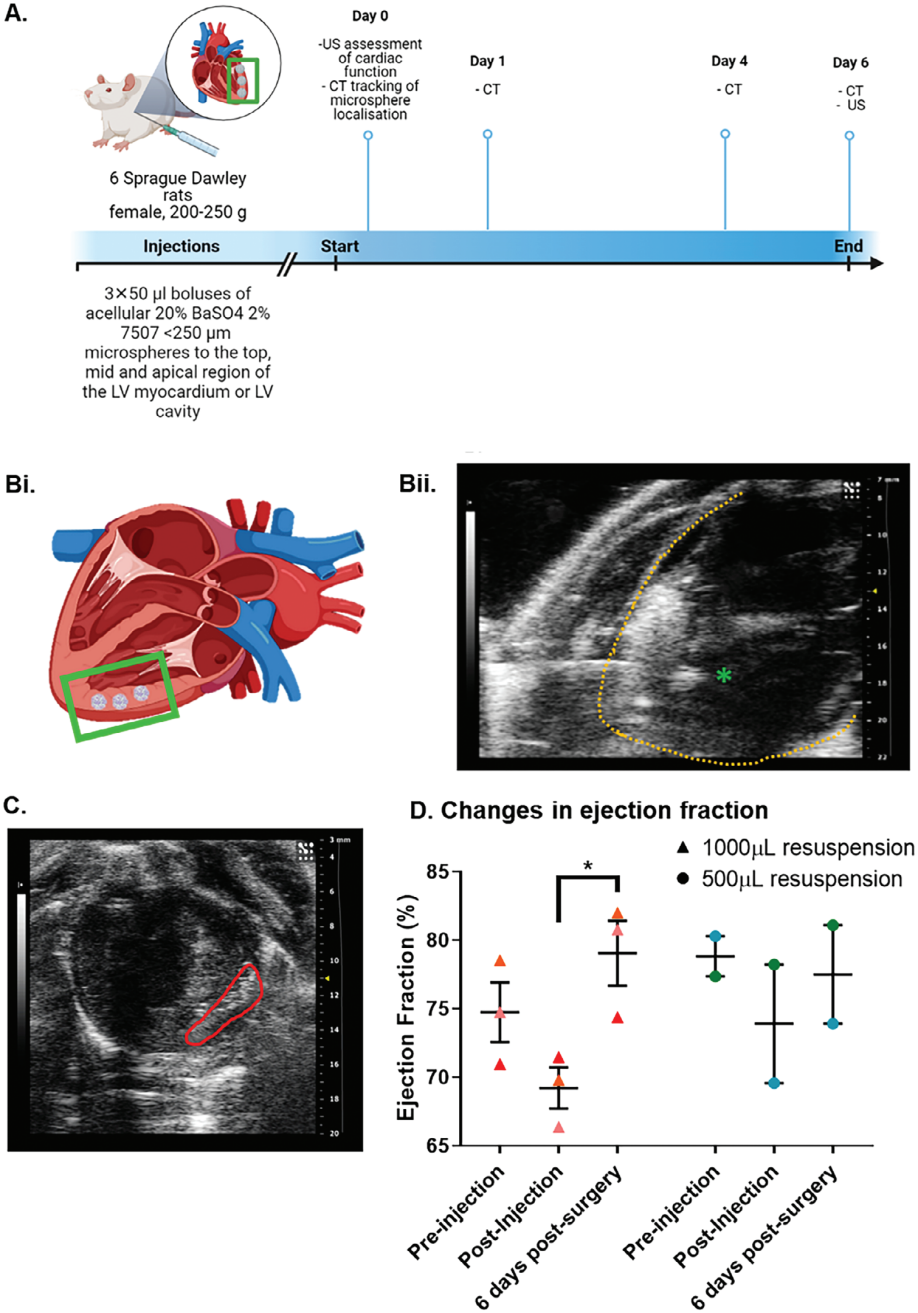
Tracking cardiac delivery of labelled microspheres in vivo. A) Timeline of the delivery protocol for acellular BaSO_4_‐loaded microspheres into a rat model. B) Targeted localization of the microspheres in the left ventricular myocardium and needle localization under ultrasound guidance. Endocardium represented by yellow dotted line and tip of needle represented by green asterisk. C) Short axis ultrasound scan of the mid‐section of a heart post‐intramyocardial injection of microspheres. D) Assessment of cardiac function following implantation of acellular BaSO_4_ microspheres. Cardiac function was measured by ejection fraction, before and after microspheres injection. Data are presented as mean ±SD. The significance of the data was calculated by Two‐way ANOVA with Tukey's post‐hoc correction. (n = 2‐3, * P = 0.0419).

As a proof‐of‐principle study, we used µCT to investigate on‐target delivery and retention of acellular 20% BaSO_4_‐loaded microspheres in the myocardium of rats after ultrasound‐guided transthoracic intramyocardial injections (**Figure** **6**A).

6 female Sprague Dawley rats 200—250 g were allocated into two groups to receive ultrasound‐guided injections of 20% BaSO_4_‐loaded microspheres. Rats received a pre‐injection ultrasound assessment of cardiac function, followed by ultrasound‐guided intramyocardial injections (3 boluses of 50 µL) in the top, mid, or apical region of the LV myocardium (Figure 6B). and a post‐injection assessment scan.

Movies of ultrasound scans pre‐, during‐, post‐injection and 3D reconstructions were acquired. The needle tip could be visualized and advanced into the myocardium. Real‐time ultrasound acquired during intra‐myocardial injections of microspheres suggested successful delivery into the myocardial wall as indicated by the presence of hyperenhancement of the ultrasound image at the site of injection (Figure 6C).

Administration of two different microsphere doses did not adversely affect cardiac function, as measured by ejection fraction (Figure 6D). In the low dose group (1000 µL resuspension), the significantly lowered ejection fraction post‐injection (mean 69.22% ±1.50 SD, n = 3, P = 0.0419, two‐way ANOVA, Tukey's post‐hoc correction) was due to the depression of cardiac contractility from the length of the anesthesia. At the post‐injection time point, the animals had been kept under anesthesia for a longer time (30–45 min), compared to pre‐injection and post‐recovery (10–15 min).

Additional measurements of heart function were also acquired and included in Figure S9 (Supporting Information).

**Figure 7:**
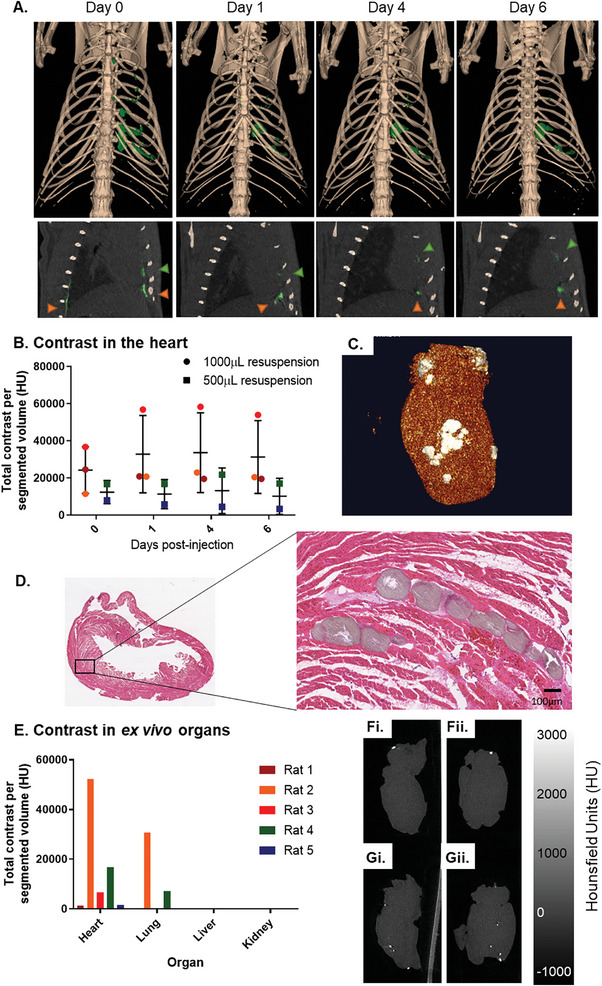
Tracking and retention of the microspheres in the heart. A) Representative sagittal cross‐section and volume rendered 3D reconstruction of CT scans show microsphere signal in green in the heart and outside of the heart. Microsphere retention in the heart was confirmed by C) µCT 3D volume‐rendered reconstruction of the *ex vivo* heart and D) histological analysis. E) Quantification of off‐target distribution of BaSO_4_‐loaded microspheres in ex vivo organs and representative brain µCT cross sections of F) control and G) non‐recovered animals.

CT was performed on days 0, 1, 4, and 6 (**Figure** **7**A) and the contrast generated by the microspheres within the heart, indicated in green or by green arrows, was quantified. Both groups showed sustained signal attenuation in the heart over the 6‐days time course (Figure 7B), suggesting that the majority of the microspheres were retained in the target tissue (Figure S10, Supporting Information).

Despite the retention of contrast, there were differences in signal between animals. The microspheres injected in rat three gave the highest signal despite receiving a low dose. The difference in signal is attributed to the challenges of the delivery technique; injecting into an area that is 2–3 mm thick under the guidance of ultrasound is difficult, particularly in a beating heart, leading to variability in both delivery and retention. Ex vivo CT and µCT reconstructions confirmed the exact location of the microspheres in the ex vivo heart (Figure 7C) and supported by histological analysis (Figure 7D).

Observations across whole body CT showed signs of off‐target delivery. Representative cross sections in Figure 7A show microspheres located outside the heart by orange arrows. Ex vivo CT cross sections of explanted organs also confirmed that a low level of microspheres was visualized in off‐target organs including lung and liver. Quantification of the contrast generated from ex vivo organs showed patterns of distribution consistent with in vivo data (Figure 7E). The majority of contrast produced by the microspheres was localized to the heart, followed by lower contrast in the lungs and trace amounts in the kidney and liver.

The importance of trackable scaffolds for detecting off‐target engraftment was evidenced in Figure 7F,G, with a low level of microspheres detected in the brain of one rat. This animal was sacrificed and ex vivo brain CT cross sections showed that BaSO_4_ positive material was present in the brain (Figure S11, Supporting Information).

## 3 Discussion

### 3.1 The Requirement for Trackable Microspheres

Targeted delivery and retention are essential requirements for implantable tissue‐engineered products. Improved methods to confirm the correct location of the implanted product and its retention or distribution thereafter would be useful in optimizing such therapies.^[^ ^25^, ^26^, ^27^ ^]^ To date, several approaches have been developed to directly track grafted cells using reporter genes,^[^ ^33^, ^34^, ^35^ ^]^ nanoparticles, and other chemical labeling agents.^[^ ^31^, ^36^, ^37^, ^38^, ^39^, ^40^ ^]^ However, these have limitations including time and facilities required for clinical grade cell labeling, potential alterations to cell phenotype and viability, and short‐term retention of contrast within the donor cells. An alternative approach is to directly label the tissue engineering scaffold material to generate an “off‐the‐shelf” approach^[^ ^41^, ^42^, ^43^ ^]^ that avoids direct cell modifications and reduces impact on cell phenotype or viability. To address this need, an injectable scaffold consisting of highly porous microspheres intended for delivery of cells was modified to contain the CT contrast agent barium sulfate (BaSO_4_).

### 3.2 Generating Distinguishable Whole‐Body Attenuation

The fabrication of injectable microspheres has been previously established and well characterized,^[^ ^44^, ^45^, ^46^ ^]^ but this paper describes the simple addition of BaSO_4_ to render the microspheres trackable. BaSO_4_ was embedded in the microspheres during fabrication to produce 2–20% (w/v) BaSO_4_‐loaded microspheres and contrast was measured against control microspheres without BaSO_4_. 2–6% BaSO_4_‐loaded microspheres produced mean contrast higher than control microspheres, but below the contrast level of soft tissue. Hence, BaSO_4_ loading was increased to 20% which generated 2024 HU, a value above that of soft tissue (100 HU) and bone (1000 HU).^[^ ^47^ ^]^ In vitro release studies confirmed that contrast generated by the BaSO_4_‐loaded microspheres remained stable up to 14 days, suggesting that the microspheres would be distinguishable in vivo over several weeks.^[^ ^48^, ^49^, ^50^ ^]^

### 3.3 In Vivo Microsphere Detection

To determine whether BaSO_4_‐loaded microspheres could be detected and tracked longitudinally in vivo, the microspheres were injected into mouse hindlimbs and µCT scanned at 0, 7, and 14 days after implant. Microspheres were clearly visible at all time‐points and volume rendering showed no change in graft volume or contrast, suggesting stable embedding and no degradation of the microspheres. This could be a useful tool for alternative applications, such as revascularization in hindlimb ischaemia, where microspheres could target the delivery of slow releasing drugs, bioactive molecules, and/or cell therapies.^[^ ^51^, ^52^ ^]^

We next investigated whether BaSO_4_‐loaded microspheres could provide information on the efficiency of cardiac delivery, an organ where cell and scaffold retention remain poor because of the dynamic nature of the heart.^[^ ^6^, ^16^, ^53^, ^54^ ^]^ Intramyocardial cell delivery has therapeutic superiority to intravenous infusion or epicardial injections,^[^ ^55^, ^56^ ^]^ but typically requires invasive open‐chest surgery with associated risks.^[^ ^55^, ^57^, ^58^ ^]^ Ultrasound guided close‐chest injections offer a minimally invasive approach for delivering therapies but are challenging to perform and require preclinical validation. Here we evaluated ultrasound‐guided injections by delivering acellular BaSO_4_‐loaded microspheres to the rat myocardium and quantified on‐ and off‐target retention using whole body µCT.

In vivo cardiac implantation of the microspheres progressed without fatalities and the animals recovered without side effects. Assessment of cardiac function immediately and 6 days after injection showed no significant changes in ejection fraction, suggesting that microspheres implantation was not detrimental to myocardial contraction.

In vivo whole body and ex vivo excised organ CT revealed BaSO_4_‐loaded microspheres could be tracked for up to 6 days after intra‐myocardial injections, with the majority of microspheres retained in the heart. However, differences in myocardial signal attenuation between animals was apparent, highlighting the challenge of consistent delivery to the heart and the importance of being able to correlate initial injection success with the ultimate functional outcome.

Given the high radiopacity of the microspheres, we tested whether single BaSO_4_‐loaded microspheres could be detected using in vitro µCT. Through comparison of light micrographs and µCT cross sections we demonstrated that 20% BaSO_4_‐loaded microspheres were distinguishable and trackable at single microsphere resolution. This approach could be of value in optimizing safe and effective modes of delivery by facilitating whole‐body distribution studies. Using this approach, we identified microspheres in off‐target sites, including lung, kidney, and liver. As expected, the lungs were the most common off‐target delivery site, as any microspheres escaping the myocardium and entering the circulation would become embedded in smaller pulmonary vessels, as has previously been demonstrated with cell infusions.^[^ ^38^ ^]^ In one animal, we detected what appeared to be four BaSO_4_ microspheres in the vasculature of the brain (Figure S12, Supporting Information). This result highlights how our approach could be useful in detecting potentially detrimental off‐target engraftment.

Barium labeled biomaterials are being developed for physiological imaging,^[^ ^59^ ^]^ biomaterial imaging,^[^ ^49^, ^60^ ^]^ and cell tracking.^[^ ^61^, ^62^ ^]^ Barium can be toxic particularly when in soluble forms.^[^ ^63^ ^]^ However, insoluble barium compounds, such as BaSO_4_ are generally non‐toxic.^[^ ^63^ ^]^ BaSO_4_‐loaded microspheres were not cytotoxic to MSC culture, unlike BaSO_4_ solutions. To further reduce toxicity concerns, BaSO_4_‐loaded microspheres could be encapsulated in alginates in order to reduce the rate of BaSO_4_ particle dissolution off the microspheres.^[^ ^64^, ^65^ ^]^ To our knowledge, there is no existing literature reporting the delivery of CT trackable microcarriers intramyocardially, but the study by Kedziorek et al. used alginate‐poly‐L‐lysine alginate microcapsules embedded with 5%, 10%, 30%, or 70% (w/v) BaSO_4_ for MSC delivery to the hindlimb.^[^ ^24^ ^]^ The proposed 10% BaSO_4_‐loaded microcapsules did not produce a contrast signal above the contrast of bone, unlike our 20% BaSO_4_‐loaded microspheres.^[^ ^24^ ^]^

### 3.4 In Vitro Microsphere Cytotoxicity

As the microspheres are designed as a substrate for transplantation of cells, we tested whether 20% BaSO_4_‐loaded microspheres were biocompatible and supported cellular attachment and proliferation in vitro. The microspheres were cellularized with GFP+ Luciferase+ murine MSC to obtain a tissue engineered construct compatible with multi‐modal imaging consisting of independently trackable scaffold and cellular components. MSCs proliferated and remained viable on BaSO_4_‐loaded and control microspheres. The difference in luminescence signal of MSC on BaSO_4_‐loaded microspheres compared with control microspheres was attributed to the difference in microsphere topography, diameter, and surface area after wetting (Figure S13, Supporting Information). The 20% BaSO_4_‐loaded microspheres did not shrink to the same extent of the control microsphere after wetting, which resulted in a sixfold difference of surface area for cell attachment and proliferation dynamics (Figure S13C, Supporting Information).

Transwell culture of MSC with BaSO_4_ loaded microspheres and matching concentrations of BaSO_4_ solutions confirmed that BaSO_4_ loaded in the microspheres was not cytotoxic. BaSO_4_ solutions showed direct MSC cytotoxicity, but loading of BaSO_4_ in the microspheres overcame the cytotoxicity. Luminescence of MSC on control and BaSO_4_ loaded microspheres was comparable. After peak luminesce was reached at day 7, the signal decreased in accordance with typical MSC growth dynamics, where cell number increased linearly, followed by exponential growth and a final plateau/ decline.^[^ ^66^ ^]^

### 3.5 In Vivo Longitudinal Multimodal Imaging

Luciferase expressing mouse MSCs were injected into mouse hindlimbs either in suspension or on BaSO_4_‐loaded microspheres. MSC delivered in suspension produced a brighter luminescent signal, which was expected in an immortalized cell line that is anchorage independent, and therefore less susceptible to anoikis.^[^ ^67^ ^]^ This experiment was a proof of principle study but paves the way for microsphere‐assisted delivery of cardiac regenerative and anchorage‐dependent cells, such as iPSC‐cardiomyocytes.

By injecting luciferase expressing MSC on trackable microspheres we were able to obtain simultaneous information on cell location and viability in relation to the biomaterial. We combined whole‐body 3D‐volume rendered BLI acquisitions with µCT reconstructions to colocalize donor cells and microspheres studied longitudinally in vivo. MSCs remained in close proximity to the microspheres when injected into the uninjured hindlimb muscle. However, future applications in injury models may be able to use this multimodal approach to monitor and optimize cell migration from the biomaterial toward the site requiring regeneration.

Although BLI tracking of cell therapy is not clinically applicable it is a useful tool for pre‐clinical investigations. In future studies, PET and SPECT reporter genes could be substituted for luciferase to generate a more clinically relevant imaging approach.^[^ ^68^, ^69^, ^70^, ^71^, ^72^, ^73^ ^]^ The routine combination of PET/SPECT with CT could then yield volumetric information on cell and biomaterial location and accurate assessment of transplantation efficiency.

## 4 Conclusion

For cardiac regeneration, the microspheres produced here have benefits over previously used hydrogel and cardiac patch approaches. Compared to hydrogels, they provide a substrate that permits robust surface attachment and patterning of cells before injection; whilst compared with patches, they are injectable, allowing minimally invasive delivery and dispersal throughout the myocardium. Additionally, microspheres could be applied to other cell therapies; functionalized to encapsulate bioactive molecules, such as slow releasing survival or angiogenic factors^[^ ^74^, ^75^ ^]^; or used for bulking or wound filler materials (TIPS Microspheres for Perianal Fistula NCT03707769).^[^ ^76^ ^]^ All these applications would benefit from non‐destructive, longitudinal studies, and quantitative analysis of microsphere distribution after delivery. Our data indicate that BaSO_4_‐loaded microspheres offer a novel tool for optimizing graft delivery, biomaterial formulation, and efficacy of regenerative treatments.

## 5 Experimental Section

### Fabrication of Implantable Microspheres

Implantable microspheres were prepared via thermally induced phase separation (TIPS) of a polymer solution, as described previously by Foong et al.,^[^ ^44^ ^]^ Blaker et al.^[^ ^46^ ^]^ and Blaker, Knowles, and Day.^[^ ^45^ ^]^ To prepare the polymer solution of 2% (w/v) PLGA, 0.8 g PURASORB PDLG 7507, 75:25 DL‐lactide/glycolide copolymer (Corbion Biomaterials) was dissolved in 40 mL dimethyl carbonate (DMC) (anhydrous >99%, D152927, Sigma Aldrich). Samples were mixed using magnetic stirring overnight at room temperature (20 °C).

For the preparation of microspheres loaded with barium sulfate (BaSO_4_) (10 442 031, Fisher Scientific), 0.8, 2.4, and 8 g BaSO_4_ were added to 40 mL of the 2% polymer solution and magnetically stirred for an additional 1 h to obtain 2%, 6%, and 20% BaSO_4_ (w/v) polymer solutions.

The polymer solutions were passed into a Var D Nisco encapsulation unit (Nisco Engineering) and ejected through a sapphire tipped nozzle with a 150 µm opening using a syringe pump at a flow rate of 2.2 mL min^−1^. The nozzle vibrated at a frequency of 1.80 kHz with 70% frequency amplitude. The resulting polymer droplets were collected in liquid nitrogen to induce phase separation. The samples underwent lyophilization for 18 h. Batches of the lyophilized microspheres were sieved to <250 µm and 250–355 µm diameter.

### Ultrastructural Imaging Using Scanning Electron Microscopy (SEM)

The microspheres were mounted on aluminum discs using adhesive carbon tabs, coated with 1–2 nm of gold/palladium compound for 3 min in the Argon chamber of a high‐resolution ion beam coater (Gatan model 681). The microspheres were then analyzed with Hitachi S3400N scanning electron microscope at a range of magnifications (x400 – x5000).

Energy Dispersive X‐ray Spectroscopy (EDS) elemental mapping was performed on the same microscope.

### Pre‐Conditioning of Microspheres

20.00 mg of microspheres or 46.87 mg of 2%,6% and 20% BaSO_4_‐loaded microspheres (normalized to microsphere count) were added to the wetting solution (5 mL DMEM (Gibco) supplemented with 10% FBS (Gibco) and 1 mL 70% Ethanol (E10600/05, Fisher Scientific)), vortexed for 10–15 s and incubated in a hybridization oven for 72 h at 37 °C under constant rotation at 30 rpm.

### In Vitro Microsphere Stability and Contrast Retention

0.1 mL of preconditioned microspheres were transferred into 0.2 mL PCR tubes (BR781332, Merck) and 150 µl of DMEM (D5796, Sigma) supplemented with 10% FBS was added to each tube. The samples were incubated at 37 °C 5% CO2 for 14 days. The release of contrast from the microspheres was assessed by the collection of spent media. The media was replaced with 150 µl of fresh DMEM. The retention of contrast in the microspheres as well as the release of contrast in the media was assessed at day 0,1,2,3,4,5,6,7,10 and 14 by µCT scanning.

### Mesenchymal Stem Cell Culture

All experiments were performed using Luciferase and GFP expressing D1 ORL UVA (ATCC CRL‐12424™) mouse mesenchymal stromal cells (a kind gift from Dr Arthur Taylor, University of Liverpool). Cells were maintained in DMEM and 10% FBS.

Cell count and viability was determined using a NucleoCounter NC‐200 automated cell counter (ChemoMetec) according to manufacturer's instructions.

### Attachment of MSC to Microspheres

The pre‐conditioned microspheres were washed with DPBS and transferred into a 24 well‐low bind cell culture plate (CLS3527, Sigma). 5 × 10^5^ MSC were suspended in 1 mL supplemented DMEM and added to each well containing the microspheres. The plate was incubated at 37 °C 5% CO_2_ on a plate shaker, shaking intermittently for 10 s every hour at low speed (≈50 rpm), for 24 h.

### Microscopy

Cellularized microspheres were stained and imaged in 8 well chambered coverglass (155409PK, Thermo Fisher). Live Dead staining was performed using LIVE/DEAD™ Viability/Cytotoxicity Kit, for mammalian cells (L3224, Invitrogen) according to the manufacturer's instructions. To visualize the distribution of cell attachment, the cellularized samples were fixed using 4% paraformaldehyde solution for 10 min at room temperature (20 °C), permeabilized with 0.1% Triton (T8787, Sigma‐Aldrich) in DPBS, for 5 min at room temperature (20 °C) and blocked for 30 min at room temperature (20 °C) using a 1% BSA solution in DPBS. The distribution of cell attachment on the microspheres was evaluated using Phalloidin 633 (A12380, Thermo Fisher) and NucBlue™ Fixed Cell Stain (R37606, Thermo Fisher) at 1:40 and 1 drop:1 mL concentration respectively.

Confocal microscopy was performed using Zeiss LSM 980 confocal with Airyscan 2 equipped with Zen Blue software. Fluorescent microscopy was done using an Evos XL (Thermo Fisher). Images were processed using Fiji software.

### XTT Assay

MSC laden microspheres were plated in 24‐ well low bind cell culture plates in triplicates. For the standard curve, MSC was plated in 24‐well plates at concentrations of 8 × 10^2 –^ 5 × 10^3^ cells per well, in triplicates. The assay was performed using Cell Proliferation Kit II (XTT) (11 465 015 001, Roche). The absorbance of all samples was measured at 475 nm and referenced at 675 nm, on a Versamax plate reader (210 988, Biomax). A standard curve was prepared by plotting the average corrected 475 nm reading of each cell standard against its concentration. The standard curve was used to interpolate the cell number on the microsphere samples.

### BaSO_4_ Cytotoxicity

1 × 10^4^ MSC was suspended in 1 mL supplemented DMEM, added into the well of a 6.5 mm Transwell plate with 8.0 µm Pore Polycarbonate Membrane Insert (3422, Corning), and cultured overnight. The direct cytotoxicity of BaSO_4_ was assessed by making 2%, 6%, and 20% (w/v) solutions of BaSO_4_ in DMEM. 54.5 µL of BaSO_4_ solution (equiv. weight of 46.87 mg of microspheres) was added into the membrane insert. The indirect cytotoxicity of BaSO_4_‐loaded microspheres was assessed by adding pre‐conditioned control, 2%, 6%, and 20% BaSO_4_‐loaded microspheres into the membrane insert. Samples were cultured overnight and cytotoxicity was measured by BLI at days 1,2,3,4,5,6,7,10, and 14 post‐ BaSO_4_ exposure. Supporting light microscopy images of cell morphology were also acquired.

### Bioluminescent Imaging (BLI)

BLI imaging was performed using an IVIS Spectrum (In vivo Imaging System, PerkinElmer). For luciferase‐based proliferation assay and cytotoxicity studies,10 µL containing 300 µg mL⁻^1^ D‐luciferin (Promega) was added to each well containing MSC laden microspheres and BLI images were acquired 10 min after luciferin injection using auto exposure time. A circular region of interest (ROI) was placed over each well for quantification.

For in vivo BLI, mice from were anaesthetized and in vivo BLI was performed in 2 h, and 1, 4, 7 and 14 days after injection. The mice were injected intraperitoneally with 75 mg k^−1^g D‐luciferin in 200 µL of PBS. Sequential BLI images were acquired 5 min after luciferin injection using auto exposure time with 3 min delay between two consecutive acquisitions. An oval ROI was placed over the hindlimb on the first image and subsequently pasted over every new image acquired until all ROIs reached their maximum intensity.

The total signal in the ROI was quantified as total flux (photons/s) by using Living Image software (PerkinElmer). Representative images were presented using radiance (the number of photons per second that leave a square centimeter of tissue and radiate into a solid angle of one steradian (sr) = p/sec/cm^2^/sr) as color scale.

### Ethics

All animal studies were approved by the University College London Biological Services Ethical Review Committee and performed with UK Home Office approval (PPL 70/8709 and PP1692884). Animal work conformed to the UK Animals (Scientific Procedures) Act, 1986 and Directive 2010/63/EU of the European Parliament. Signs of stress and the general wellbeing of the animals were observed throughout the study by monitoring of body weight and behavior.

### Hindlimb Implantation of BaSO_4_ Loaded Microspheres

10 female BALB/c mice, 26 weeks old, (Charles River) were anesthetized with 1.5% – 2.5% isoflurane (PRI001325‐EA, SAS) in 1.5 – 2 L min^−1^ oxygen flow. 10 animals received injections of 50 µL of, 1.36 × 10^6^ MSC suspended in 1 mL 60% Granugel (n = 5) or cellularised microspheres, consisting of 46.87 mg of wetted 20% BaSO_4_‐loaded microspheres (<250 µm) suspended in 1 mL 60% Granugel (n = 5), into the left flank. Following the injections, the mice were placed in heated recovery chambers for 1 h, transferred back into their cages, and monitored for 7–14 days. The animals were sacrificed using Schedule 1 approved methods of heart removal and confirmation of death by cervical dislocation.

### Intramyocardial Injections of BaSO_4_ Loaded Microspheres

Six female Sprague Dawley rats 200—250 g (Charles River) were anesthetized with 1.5% – 2.5% isoflurane in 1.5 – 2 L min^−1^ oxygen flow. Each rat was placed in a supine position on the surgery table and connected using a face mask to a continuous flow of isoflurane and oxygen. A 2 × 4 cm patch was shaved on the left section of the ribcage. The animal and anesthetic face mask were transferred and secured to the ultrasound table. Cardiac assessment ultrasound scans prior to microsphere administration were collected using FujiFilm Visualsonics Vevo2100.

Rats underwent ultrasound‐guided intramyocardial injections of 3 boluses containing 50 µL of, 46.87 mg of wetted 20% BaSO_4_ microspheres suspended in either 500 µL or 1000 µL of 60% Granugel, into the basal, mid, and apical region of the left ventricle. Cardiac function was assessed immediately after and 6 days after injection using m‐mode ultrasound to calculate ejection fraction, fractional shortening, left‐ventricular mass, and left‐ventricular volume. The animals were sacrificed using Schedule 1 approved methods and the organs were fixed by cardiac perfusion fixation. The hearts were exposed, the pleural cavity was opened and 20 mL potassium chloride in saline (30 mM KCl, 0.9% w/v (154 mM NaCl) saline) was injected into the left ventricle, followed by 20 mL 4% PFA injection into the left ventricle for whole body perfusion. The heart, lung, liver, kidney, and brain were dissected out, fixed in 4% PFA overnight, and embedded in paraffin for H&E staining.

### Computed Tomography Imaging of BaSO_4_‐Loaded Microspheres

Computed tomography (CT) images of microspheres loaded with BaSO_4_ were acquired using a µCT scanner (Perkin Elmer Quantum GX2) with X‐ray source set at 90 kVp and 88 µA, with a large field of view (FOV = 72 mm, recon 72 mm, standard 2 min scan time). X‐ray projections were reconstructed to give CT images with 144 µm isotropic voxel resolution using 3D Viewer (Perkin Elmer). ROIs were drawn manually and histograms were then produced showing the distribution of signal intensities in Hounsfield units (HU) across voxels in the region of interest, as well as mean HU and standard deviation.

### In Vivo Whole Animal µCT

µCT images of the injected animals were acquired to monitor microsphere distribution at days 0, 7, and 14 post‐injection. The animals were anesthetized and laid in a supine position in the µCT scanner (Perkin Elmer Quantum GX2). Whole‐body CT images were acquired with X‐ray source set at 90 kVp and 88 µA, with a large field of view (FOV = 72 mm, recon 72 mm, whole body 3 × 24 seconds scan time). Higher resolution CT images of the hindlimbs were acquired with X‐ray source set at 90 kVp and 88 µA, with a medium field of view (FOV = 36 mm, recon 36 mm, standard 2 min scan time). X‐ray projections were reconstructed to give CT images with 72 µm isotropic voxel resolution. Following the scan, the animals were placed in heated recovery chambers for 30 min and transferred back to their cages.

### Ex Vivo Organ µCT

CT images of the ex vivo organs were acquired immediately after dissection. The organs were dabbed dry from any liquid and CT images were acquired using the µCT scanner, with X‐ray source set at 90 kVp and 88 µA, with a medium field of view (FOV = 36 mm, recon 36 mm, high resolution 10 min scan time).

### Image Processing

Image segmentation was performed using the Analyze 12.0 software (AnalyzeDirect) using a combination of the “Semi‐automatic” and “Manual Technique” settings. A 3D rendering of the implanted microspheres and bone was generated, from which volumes and signal intensities were calculated.

### 3D BLI Co‐Registration with Whole Body µCT

Localization of the MSC to the injected microspheres within the hindlimb was determined by co‐registering µCT and 3D BLI data. The animals were anesthetized and injected intraperitoneally with 75 mg k^−1^g D‐luciferin in 200 µL of PBS and laid in a supine position in mouse imaging shuttle to immobilize the animal in a stable position. 3D tomographic images were acquired 15 min after luciferin injection, by acquiring 2D bioluminescent surface radiance images with emission filters 560–640 nm. The anesthetizedanimal in the imaging shuttle was transferred to the µCT to acquire anatomical reference data, using an X‐ray source set at 90 kVp and 88 µA, with a large field of view (FOV = 72 mm, recon 72 mm, whole body 3 × 24 seconds scan time). Combined whole‐body µCT and DLIT images were reconstructed in Living Image software.

### Statistical Analysis

Data reported was collected from technical and biological replicates with samples sizes indicated in figure legends. Data are presented as mean and, standard deviation (±SD). Prior to statistical analysis, the data was first tested for normality through a Shapiro‐Wilk test. Statistical significance for the difference between experimental groups was determined using a Student's t‐test. Analysis of statistical significance for two groups or more was performed using ANOVA followed by post‐hoc test. The data were plotted using GraphPad Prism 8 Software (GraphPad Holdings LLC).

## Conflict of Interest

The authors declare no conflict of interest.

## Supporting information

Supporting Information

Supporting Information Video 1

Supporting Information Video 2

Supporting Information Video 3

## Data Availability

Data supporting this study are included within this article and supporting materials. Full data are available from the corresponding author upon reasonable request.

## References

[1] T. J. Cahill , R. P. Choudhury , P. R. Riley , Nat. Rev. Drug Discov. 2017, 16, 699. 28729726 10.1038/nrd.2017.106

[2] R. Bolli , M. Solankhi , X. L. Tang , A. Kahlon , Cardiovasc. Res. 2022, 118, 951. 33871588 10.1093/cvr/cvab135 PMC8930075

[3] M. A. Laflamme , K. Y. Chen , A. V. Naumova , V. Muskheli , J. A. Fugate , S. K. Dupras , H. Reinecke , C. Xu , M. Hassanipour , S. Police , C. O'Sullivan , L. Collins , Y. Chen , E. Minami , E. A. Gill , S. Ueno , C. Yuan , J. Gold , C. E. Murry , Nat. Biotechnol. 2007, 25, 1015. 17721512 10.1038/nbt1327

[4] E. Karbassi , A. Fenix , S. Marchiano , N. Muraoka , K. Nakamura , X. Yang , C. E. Murry , Nat. Rev. Cardiol. 2020, 17, 341. 32015528 10.1038/s41569-019-0331-x PMC7239749

[5] K. A. Gerbin , C. E. Murry , Cardiovasc. Pathol. 2015, 24, 133. 25795463 10.1016/j.carpath.2015.02.004 PMC4430350

[6] J. Li , S. Hu , D. Zhu , K. Huang , X. Mei , B. López de Juan Abad , K. Cheng , J. Am. Heart Assoc. 2021, 10, 1. 10.1161/JAHA.120.020402 PMC8174178 33821664

[7] T. Eschenhagen , M. Didié , F. Münzel , P. Schubert , K. Schneiderbanger , W. H. Zimmermann , Basic Res. Cardiol. 2002, 97, 1146. 10.1007/s003950200043 12479248

[8] C. L. Hastings , E. T. Roche , E. Ruiz‐Hernandez , K. Schenke‐Layland , C. J. Walsh , G. P. Duffy , Adv. Drug Deliv. Rev. 2015, 84, 85. 25172834 10.1016/j.addr.2014.08.006

[9] J. L. Ifkovits , E. Tous , M. Minakawa , M. Morita , J. D. Robb , K. J. Koomalsingh , J. H. Gorman , R. C. Gorman , J. A. Burdick , Proc. Natl. Acad. Sci. U. S. A. 2010, 107, 11507. 20534527 10.1073/pnas.1004097107 PMC2895138

[10] J. Venugopal , R. Rajeswari , M. Shayanti , R. Sridhar , S. Sundarrajan , R. Balamurugan , S. Ramakrishna , Mater. Sci. Eng.: C 2013, 33, 1325. 10.1016/j.msec.2012.12.032 23827578

[11] V. V. Alvino , R. Fernández‐Jiménez , I. Rodriguez‐Arabaolaza , S. Slater , G. Mangialardi , E. Avolio , H. Spencer , L. Culliford , S. Hassan , L. S. Ballesteros , A. Herman , A. Ayaon‐Albarran , C. Galan‐Arriola , J. Sanchez‐Gonzalez , H. Hennessey , C. Delmege , R. Ascione , C. Emanueli , G. D. Angelini , B. Ibanez , P. Madeddu , J. Am. Heart Assoc. 2018, 7, e006727. 29358198 10.1161/JAHA.117.006727 PMC5850145

[12] M. F. Tenreiro , A. F. Louro , P. M. Alves , M. Serra , npj Regen. Med. 2021, 6, 30. 34075050 10.1038/s41536-021-00140-4 PMC8169890

[13] P. Montero , M. Flandes‐Iparraguirre , S. Musquiz , M. Pérez Araluce , D. Plano , C. Sanmartín , G. Orive , J. J. Gavira , F. Prosper , M. M. Mazo , Front. Bioeng. Biotechnol. 2020, 8, 00955. 10.3389/fbioe.2020.00955 PMC7431658 32850768

[14] X. Mei , K. Cheng , Front. Cardiovasc. Med. 2020, 7, 610364. 33330673 10.3389/fcvm.2020.610364 PMC7728668

[15] W. H. Zimmermann , I. Melnychenko , G. Wasmeier , M. Didié , H. Naito , U. Nixdorff , A. Hess , L. Budinsky , K. Brune , B. Michaelis , S. Dhein , A. Schwoerer , H. Ehmke , T. Eschenhagen , Nat. Med. 2006, 12, 452. 16582915 10.1038/nm1394

[16] T. Eschenhagen , K. Ridders , F. Weinberger , J. Mol. Cell. Cardiol. 2022, 163, 106. 34687723 10.1016/j.yjmcc.2021.10.005

[17] I. Mannhardt , K. Breckwoldt , D. Letuffe‐Brenière , S. Schaaf , H. Schulz , C. Neuber , A. Benzin , T. Werner , A. Eder , T. Schulze , B. Klampe , T. Christ , M. N. Hirt , N. Huebner , A. Moretti , T. Eschenhagen , A. Hansen , Stem Cell Rep. 2016, 7, 29. 10.1016/j.stemcr.2016.04.011 PMC4944531 27211213

[18] J. Riegler , M. Tiburcy , A. Ebert , E. Tzatzalos , U. Raaz , O. J. Abilez , Q. Shen , N. G. Kooreman , E. Neofytou , V. C. Chen , M. Wang , T. Meyer , P. S. Tsao , A. J. Connolly , L. A. Couture , J. D. Gold , W. H. Zimmermann , J. C. Wu , Circ. Res. 2015, 117, 720. 26291556 10.1161/CIRCRESAHA.115.306985 PMC4679370

[19] M. Kawamura , S. Miyagawa , S. Fukushima , A. Saito , K. Miki , S. Funakoshi , Y. Yoshida , S. Yamanaka , T. Shimizu , T. Okano , T. Daimon , K. Toda , Y. Sawa , Sci. Rep. 2017, 7, 8824. 28821761 10.1038/s41598-017-08869-z PMC5562896

[20] L. Gao , Z. R. Gregorich , W. Zhu , S. Mattapally , Y. Oduk , X. Lou , R. Kannappan , A. V. Borovjagin , G. P. Walcott , A. E. Pollard , V. G. Fast , X. Hu , S. G. Lloyd , Y. Ge , J. Zhang , Circulation 2018, 137, 1712. 29233823 10.1161/CIRCULATIONAHA.117.030785 PMC5903991

[21] E. Querdel , M. Reinsch , L. Castro , D. Köse , A. Bähr , S. Reich , B. Geertz , B. Ulmer , M. Schulze , M. D. Lemoine , T. Krause , M. Lemme , J. Sani , A. Shibamiya , T. Studemann , M. Kohne , C. von Bibra , N. Hornaschewitz , S. Pecha , Y. Nejahsie , I. Mannhardt , T. Christ , H. Reichenspurner , A. Hansen , N. Klymiuk , M. Krane , C. Kupatt , T. Eschenhagen , F. Weinberger , Circulation 2021, 143, 1991. 33648345 10.1161/CIRCULATIONAHA.120.047904 PMC8126500

[22] S. Hinderer , E. Brauchle , K. Schenke‐Layland , Adv. Healthcare Mater. 2015, 4, 2326. 10.1002/adhm.201400762 PMC4745029 25778713

[23] T. Wu , W. Liu , NPG Asia Mater. 2022, 14, 9.

[24] D. A. Kedziorek , L. V. Hofmann , Y. Fu , W. D. Gilson , K. M. Cosby , B. Kohl , B. P. Barnett , B. W. Simons , P. Walczak , J. W. M. Bulte , K. Gabrielson , D. L. Kraitchman , Stem Cells 2012, 30, 1286. 22438076 10.1002/stem.1096 PMC3653421

[25] J. P. K. Armstrong , T. J. Keane , A. C. Roques , P. S. Patrick , C. M. Mooney , W. L. Kuan , V. Pisupati , R. O. C. Oreffo , D. J. Stuckey , F. M. Watt , et al., Sci. Transl. Med. 2020, 12, aaz2253. 10.1126/scitranslmed.aaz2253 PMC7610850 33268507

[26] B. M. Helfer , V. Ponomarev , P. S. Patrick , P. J. Blower , A. Feitel , G. O. Fruhwirth , S. Jackman , L. Pereira Mouriès , M. V. D. Z. Park , M. Srinivas , et al., Cytotherapy 2021, 23, 757. 33832818 10.1016/j.jcyt.2021.02.005 PMC9344904

[27] L. Scarfe , N. Brillant , J. D Kumar , N. Ali , A. Alrumayh , M. Amali , S. Barbellion , V. Jones , M. Niemeijer , S. Potdevin , G. Roussignol , A. Vaganov , I. Barbaric , M. Barrow , N C. Burton , J. Connell , F. Dazzi , J. Edsbagge , N S. French , J. Holder , C. Hutchinson , D R. Jones , T. Kalber , C. Lovatt , M F. Lythgoe , S. Patel , P. S Patrick , J. Piner , J. Reinhardt , E. Ricci , et al., NPJ Regen Med 2017, 2. 10.1038/s41536-017-0029-9 PMC5677988 29302362

[28] D. J. Stuckey , C. A. Carr , E. Martin‐Rendon , D. J. Tyler , C. Willmott , P. J. Cassidy , S. J. M. Hale , J. E. Schneider , L. Tatton , S. E. Harding , et al., Stem Cells 2006, 24, 1968. 16627684 10.1634/stemcells.2006-0074

[29] N. Nose , S. Nogami , K. Koshino , X. Chen , R. A. Werner , S. Kashima , S. P. Rowe , C. Lapa , K. Fukuchi , T. Higuchi , Sci. Rep. 2021, 11, 10896. 34035416 10.1038/s41598-021-90383-4 PMC8149709

[30] M. Aswendt , S. Vogel , C. Schäfer , A. Jathoul , M. Pule , M. Hoehn , Neurophotonics 2019, 6, 1. 10.1117/1.NPh.6.2.025006 PMC6504011 31093514

[31] M. Zaw Thin , O. Ogunlade , J. Comenge , P. S. Patrick , D. J. Stuckey , A. L. David , M. F. Lythgoe , P. Beard , T. L. Kalber , Sci. Rep. 2020, 10, 7514. 32372054 10.1038/s41598-020-64417-2 PMC7200714

[32] A. T. Speidel , D. J. Stuckey , L. W. Chow , L. H. Jackson , M. Noseda , M. Abreu Paiva , M. D. Schneider , M. M. Stevens , ACS Cent. Sci. 2017, 3, 338. 28470052 10.1021/acscentsci.7b00039 PMC5408339

[33] P. S. Patrick , T. B. Rodrigues , M. I. Kettunen , S. K. Lyons , A. A. Neves , K. M. Brindle , Magn Reson Med 2016, 75, 1697. 25981669 10.1002/mrm.25750 PMC4832381

[34] P. S Patrick , M. I. Kettunen , S. S. Tee , T. B. Rodrigues , E. Serrao , K. N. Timm , S. McGuire , K. M. Brindle , Magn Reson Med 2015, 73, 1401. 24733406 10.1002/mrm.25254

[35] P. S Patrick , J. Hammersley , L. Loizou , M I. Kettunen , T B. Rodrigues , D.‐En Hu , S.‐S. Tee , R. Hesketh , S K. Lyons , D. Soloviev , D Y. Lewis , S. Aime , S M. Fulton , K M. Brindle , Proc. Natl. Acad. Sci. USA 2014, 111, 415. 24347640 10.1073/pnas.1319000111 PMC3890795

[36] P. S. Patrick , K. K. Kolluri , M. Zaw Thin , A. Edwards , E. K. Sage , T. Sanderson , B. D. Weil , J. C. Dickson , M. F. Lythgoe , M. Lowdell , et al., Stem cell research & therapy 2020, 11, 256. 32586403 10.1186/s13287-020-01770-z PMC7318529

[37] P. S. Patrick , L. K. Bogart , T. J. Macdonald , P. Southern , M. J. Powell , M. Zaw‐Thin , N. H. Voelcker , I. P. Parkin , Q. A. Pankhurst , M. F. Lythgoe , et al., Chem. Sci. 2019, 10, 2592. 30996974 10.1039/c8sc04895a PMC6419938

[38] M. Zaw Thin , H. Allan , R. Bofinger , T. D. Kostelec , S. Guillaume , J. J. Connell , P. S. Patrick , H. C. Hailes , A. B. Tabor , M. F. Lythgoe , et al., Nanoscale 2020, 12, 16570. 32749427 10.1039/d0nr03237a PMC7586303

[39] D. J. Stuckey , C. A. Carr , E. Martin‐Rendon , D. J. Tyler , C. Willmott , P. J. Cassidy , S. J. M. Hale , J. E. Schneider , L. Tatton , S. E. Harding , et al., Stem Cells 2006, 24, 1968. 16627684 10.1634/stemcells.2006-0074

[40] C A. Carr , D J. Stuckey , L. Tatton , D J. Tyler , S J. M. Hale , D. Sweeney , J E. Schneider , E. Martin‐Rendon , G K. Radda , S E. Harding , S M. Watt , K. Clarke , American Journal of Physiology – Heart and Circulatory Physiology 2008, 295. 10.1152/ajpheart.00094.2008 PMC2519197 18539761

[41] P. S. Patrick , J. C. Bear , H. E. Fitzke , M. Zaw‐Thin , I. P. Parkin , M. F. Lythgoe , T. L. Kalber , D. J. Stuckey , Biomaterials 2020, 243, 119930. 32171101 10.1016/j.biomaterials.2020.119930 PMC7103761

[42] B. P. Barnett , D. L. Kraitchman , C. Lauzon , C. A. Magee , P. Walczak , W. D. Gilson , A. Arepally , J. W. M. Bulte , Mol. Pharmaceutics 2006, 3, 531. 10.1021/mp060056l 17009852

[43] B. P. Barnett , A. Arepally , M. Stuber , D. R. Arifin , D. L. Kraitchman , J. W. M. Bulte , Nat. Protoc. 2011, 6, 1142. 21799484 10.1038/nprot.2011.352 PMC3193154

[44] K. S. Foong , R. Patel , A. Forbes , R. M. Day , Tissue Eng., Part C 2010, 16, 855. 10.1089/ten.TEC.2009.0599 19886803

[45] J. J. Blaker , J. C. Knowles , R. M. Day , Acta Biomater. 2008, 4, 264. 18032120 10.1016/j.actbio.2007.09.011

[46] J. J. Blaker , J. Pratten , D. Ready , J. C. Knowles , A. Forbes , R. M. Day , Aliment. Pharmacol. Ther. 2008, 28, 614. 18565160 10.1111/j.1365-2036.2008.03773.x

[47] J. Broder , Diagnostic Imaging for the Emergency Physician, Elsevier Inc, Philadelphia 2011.

[48] Q. Zhang , H. Mi , X. Shi , W. Li , S. Guo , P. Wang , H. Suo , Z. Wang , S. Jin , F. Yan , et al., Acad Radiol 2021, 28, 1072. 32553279 10.1016/j.acra.2020.05.012

[49] Y. Lian , F. Feng , X. Meng , Y. Hu , M. Huo , G. Wang , J. Li , Biomater. Sci. 2023, 4907. 37306064 10.1039/d3bm00325f

[50] O. Rabin , J. M. Perez , J. Grimm , G. Wojtkiewicz , R. Weissleder , Nat. Mater. 2006, 5, 118. 16444262 10.1038/nmat1571

[51] E. K. Hendow , M. Moazen , F. Iacoviello , L. Bozec , C. Pellet‐Many , R. M. Day , Adv. Healthcare Mater. 2020, 9. 10.1002/adhm.202000806 PMC8427471 32666663

[52] C. Simitzi , E. Hendow , Z. Li , R. M. Day , Adv. Biosyst. 2020, 4. 10.1002/adbi.202000062 PMC8425330 32511898

[53] D. Zhu , Z. Li , K. Huang , T. G. Caranasos , J. S. Rossi , K. Cheng , Nat. Commun. 2021, 12, 1. 33658506 10.1038/s41467-021-21682-7 PMC7930285

[54] J. R. Garcia , P. F. Campbell , G. Kumar , J. J. Langberg , L. Cesar , L. Wang , A. J. García , R. D. Levit , JACC Basic Transl Sci 2017, 2, 601. 30062173 10.1016/j.jacbts.2017.06.003 PMC6058920

[55] K. A. Gerbin , X. Yang , C. E. Murry , K. L. K. Coulombe , PLoS One 2015, 10, e0131446. 26161513 10.1371/journal.pone.0131446 PMC4498815

[56] S. Kadota , Y. Shiba , Curr Cardiol Rep 2019, 21. 10.1007/s11886-019-1171-3 31228011

[57] A. Chow , D. J. Stuckey , E. Kidher , M. Rocco , R. J. Jabbour , C. A. Mansfield , A. Darzi , S. E. Harding , M. M. Stevens , T. Athanasiou , Stem Cell Rep. 2017, 9, 1415. 10.1016/j.stemcr.2017.09.003 PMC5830963 28988988

[58] M. A. Laflamme , K. Y. Chen , A. V. Naumova , V. Muskheli , J. A. Fugate , S. K. Dupras , H. Reinecke , C. Xu , M. Hassanipour , S. Police , et al., Nat. Biotechnol. 2007, 25, 1015. 17721512 10.1038/nbt1327

[59] M. S. Kader , C. Weyer , A. Avila , S. Stealey , S. Sell , S. P. Zustiak , S. Buckner , S. McBride‐Gagyi , P. A. Jelliss , ACS Mater Au 2022, 2, 260. 36855388 10.1021/acsmaterialsau.1c00070 PMC9888639

[60] G. N. Schädli , H. David , A. M. de Leeuw , F. Carlson , L. Tenisch , P. Muff , M. Rubert , R. Müller , Frontiers in Materials 2022, 8, 1.

[61] D. V. Shepherd , J. H. Shepherd , S. M. Best , R. E. Cameron , J. Mater. Sci.: Mater. Med. 2018, 29, 86. 29896644 10.1007/s10856-018-6089-6 PMC5997119

[62] V. Sokolova , K. Loza , J. F. Ebel , J. Buer , A. M. Westendorf , M. Epple , Acta Biomater. 2023, 164, 577. 37019167 10.1016/j.actbio.2023.03.043

[63] D. Moffet , C. Smith , Y. Stevens , L. Ingerman , S. Swarts , L. Chappell , Agency for toxic substances and disease registry 2007, 1.

[64] N. Konduru , J. Keller , L. Ma‐Hock , S. Gröters , R. Landsiedel , T. C. Donaghey , J. D. Brain , W. Wohlleben , R. M. Molina , Part. Fibre Toxicol. 2014, 11, 1. 25331813 10.1186/s12989-014-0055-3 PMC4219084

[65] Q. Hou , D. Y. S. Chau , C. Pratoomsoot , P. J. Tighe , H. S. Dua , K. M. Shakesheff , F. R. A. J. Rose , J. Pharm. Sci. 2008, 97, 3972. 18240277 10.1002/jps.21310

[66] J. Chang , H. Lei , Q. Liu , S. Qin , K. Ma , S. Luo , X. Zhang , W. Huang , Z. Zuo , H. Fu , Y. Xia , Cell Prolif. 2012, 45, 430. 22925502 10.1111/j.1365-2184.2012.00836.x PMC6496647

[67] F. Ishikawa , K. Ushida , K. Mori , M. Shibanuma , Cell Death Dis. 2015, 6, e1619. 25611393 10.1038/cddis.2014.583 PMC4669778

[68] R. Vandergaast , S. Khongwichit , H. Jiang , T. R. DeGrado , K.‐W. Peng , D. R. Smith , S. J. Russell , L. Suksanpaisan , Cancer Gene Ther. 2020, 27, 179. 30674994 10.1038/s41417-019-0081-2 PMC7170803

[69] B. Holvoet , M. Quattrocelli , S. Belderbos , L. Pollaris , E. Wolfs , O. Gheysens , R. Gijsbers , J. Vanoirbeek , C. M. Verfaillie , M. Sampaolesi , et al., Stem Cell Rep. 2015, 5, 1183. 10.1016/j.stemcr.2015.10.018 PMC4682284 26626179

[70] Z. Love , F. Wang , J. Dennis , A. Awadallah , N. Salem , Y. Lin , A. Weisenberger , S. Majewski , S. Gerson , Z. Lee , J Nucl Med 2007, 48, 1408. 18006616 10.2967/jnumed.107.043166

[71] K. Neyrinck , N. Breuls , B. Holvoet , W. Oosterlinck , E. Wolfs , H. Vanbilloen , O. Gheysens , R. Duelen , W. Gsell , I. Lambrichts , et al., Theranostics 2018, 8, 2799. 29774076 10.7150/thno.22980 PMC5957010

[72] Z. Pei , X. Lan , Z. Cheng , C. Qin , X. Xia , H. Yuan , Z. Ding , Y. Zhang , PLoS One 2014, 9, e90543. 24608323 10.1371/journal.pone.0090543 PMC3946457

[73] N. Sun , A. Lee , J. C. Wu , Nat. Protoc. 2009, 4, 1192. 19617890 10.1038/nprot.2009.100 PMC3683546

[74] K. Paliashvili , F. Di Maggio , H. M. K. Ho , S. Sathasivam , H. Ahmed , R. M. Day , Drug Delivery 2019, 26, 1115. 31735095 10.1080/10717544.2019.1686085 PMC6882460

[75] K. Paliashvili , A. Popov , T. L. Kalber , P. S. Patrick , A. Hayes , A. Henley , F. I. Raynaud , H. U. Ahmed , R. M. Day , Advanced Therapeutics 2021, 4, 2000179. 34527807 10.1002/adtp.202000179 PMC8427470

[76] ClinicalTrials.gov, “TIPS Microspheres for Perianal Fistula” can be found under https://clinicaltrials.gov/ct2/show/NCT03707769, 2018.

