# Supplementary material for: CT‐Visible Microspheres Enable Whole‐Body In Vivo Tracking of Injectable Tissue Engineering Scaffolds
Source: Adv Healthc Mater. 2024 May 4;13(17):2303588. doi: 10.1002/adhm.202303588 (PMC11468734; doi:10.1002/adhm.202303588)
Supplement: Supplementary file 1 — Supporting Information [file ADHM-13-2303588-s003.pdf]

ADVANCED HEALTHCARE MATERIALS

Supporting Information

for *Adv. Healthcare Mater.*, DOI 10.1002/adhm.202303588

CT-Visible Microspheres Enable Whole-Body In Vivo Tracking of Injectable Tissue Engineering Scaffolds

Annalisa Bettini, Peter Stephen Patrick, Richard M. Day and Daniel J. Stuckey**

Supporting Information

CT-Visible Microspheres Enable Whole-body *In Vivo* Tracking of Injectable Tissue Engineering Scaffolds

Annalisa Bettini, P. Stephen Patrick, Richard M. Day and Daniel J. Stuckey**

Video 1: 3D reconstruction of microspheres in the hindlimb

Video 2: Co-registration of reconstructed 3D volume rendered BLI and µCT reconstructions showed cell and scaffold co-localisation in the hindlimb

Video 3: Whole body 3D reconstruction of microspheres in a rat heart

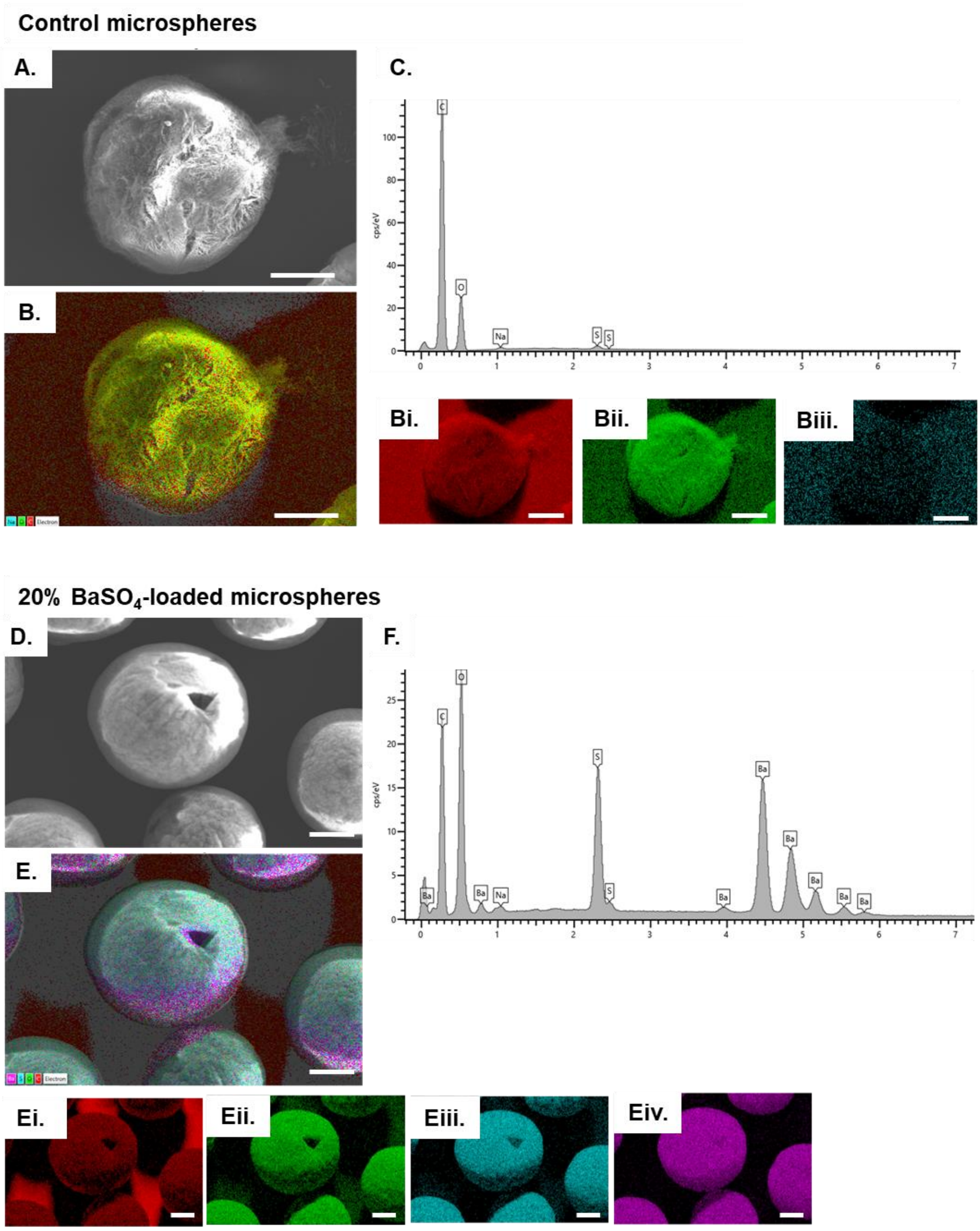

Figure S1: EDX analysis of control microspheres and 20% $BaSO_4$-loaded microspheres. (A) Electron image and (B) overlayed EDX image, showing the presence of (i) carbon in red, (ii) oxygen in green and (iii) sodium in blue. (C) Corresponding elemental analysis. Scale bar represents 100 µm. (D) Electron image and (E) overlayed EDX image, showing the presence of (i) carbon in red, (ii) oxygen in green, (iii) sulphur in blue and (iv) barium in pink. (F) Corresponding elemental analysis. Scale bar represents 100 µm.

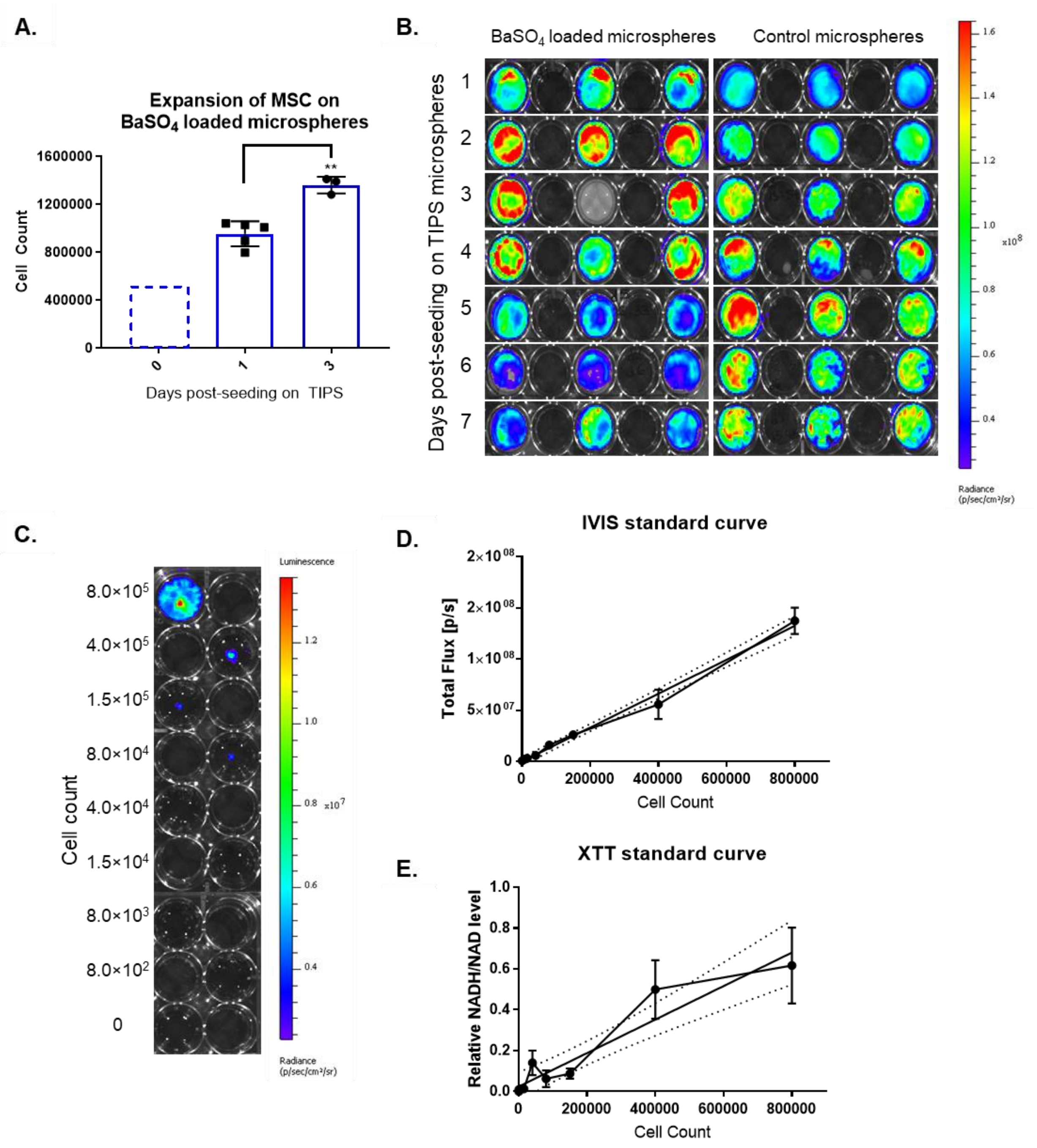

Figure S2: ***In vitro*** **characterisation of MSC culture on microspheres.** Quantification of MSC (A) proliferation, and (B) luminescence of MSC on 20% w/ $BaSO_4$ loaded- and control- microspheres. (C and D) luminescent activity correlation of MSC seeded at densities from 8.0×10^5 through 8.0×10^2 cells. (E) Quantification of luminescence activity of MSC on microspheres over 7 days. Data are presented as mean ±SD. The significance of the data was calculated by unpaired two-tailed t-test. (n=3-5, P=0.0011).

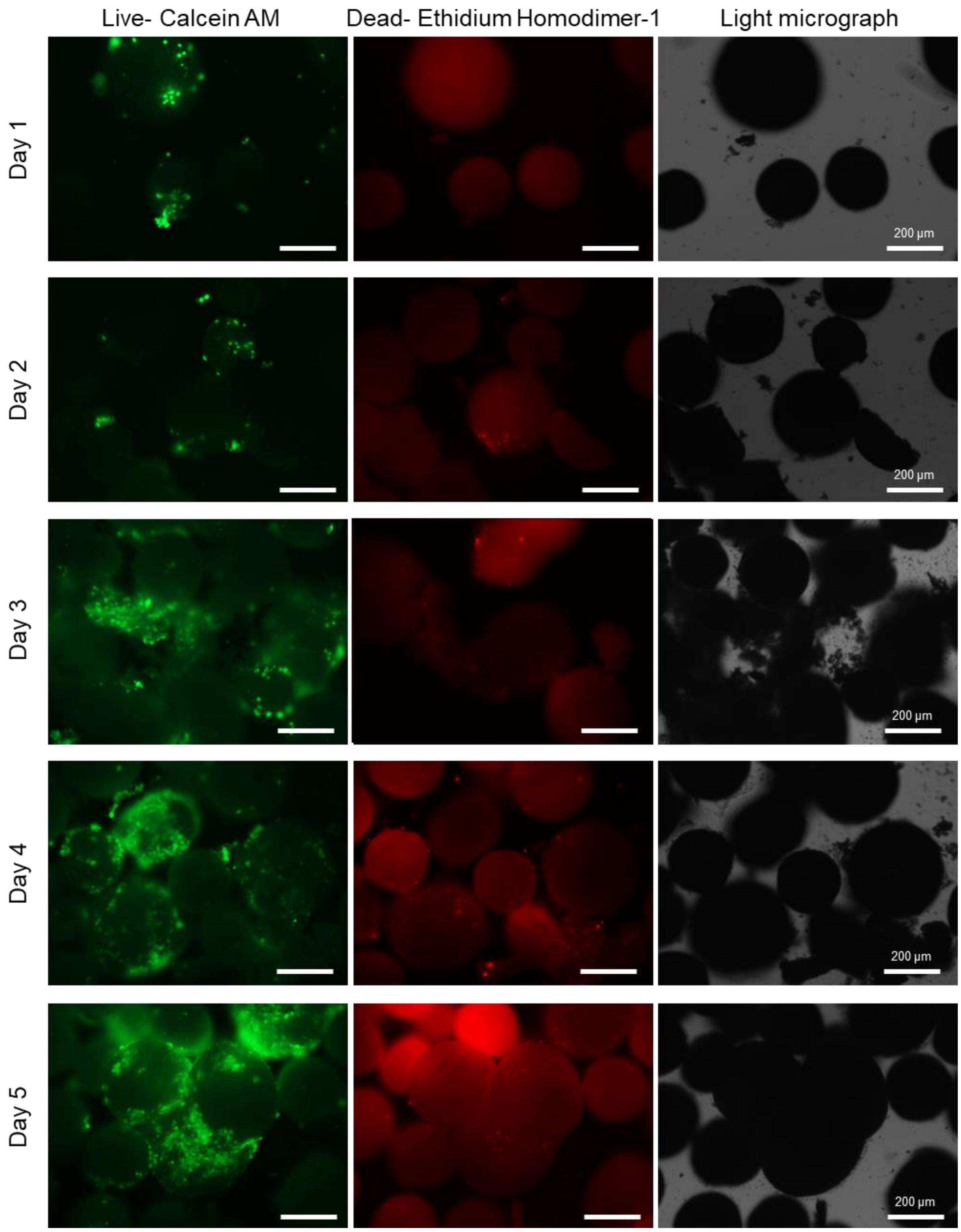

Live- Calcein AM
Dead- Ethidium Homodimer-1
Light micrograph
Day 1
Day 2
Day 3
Day 4
Day 5
200 µm
200 µm
200 µm
200 µm
200 µm

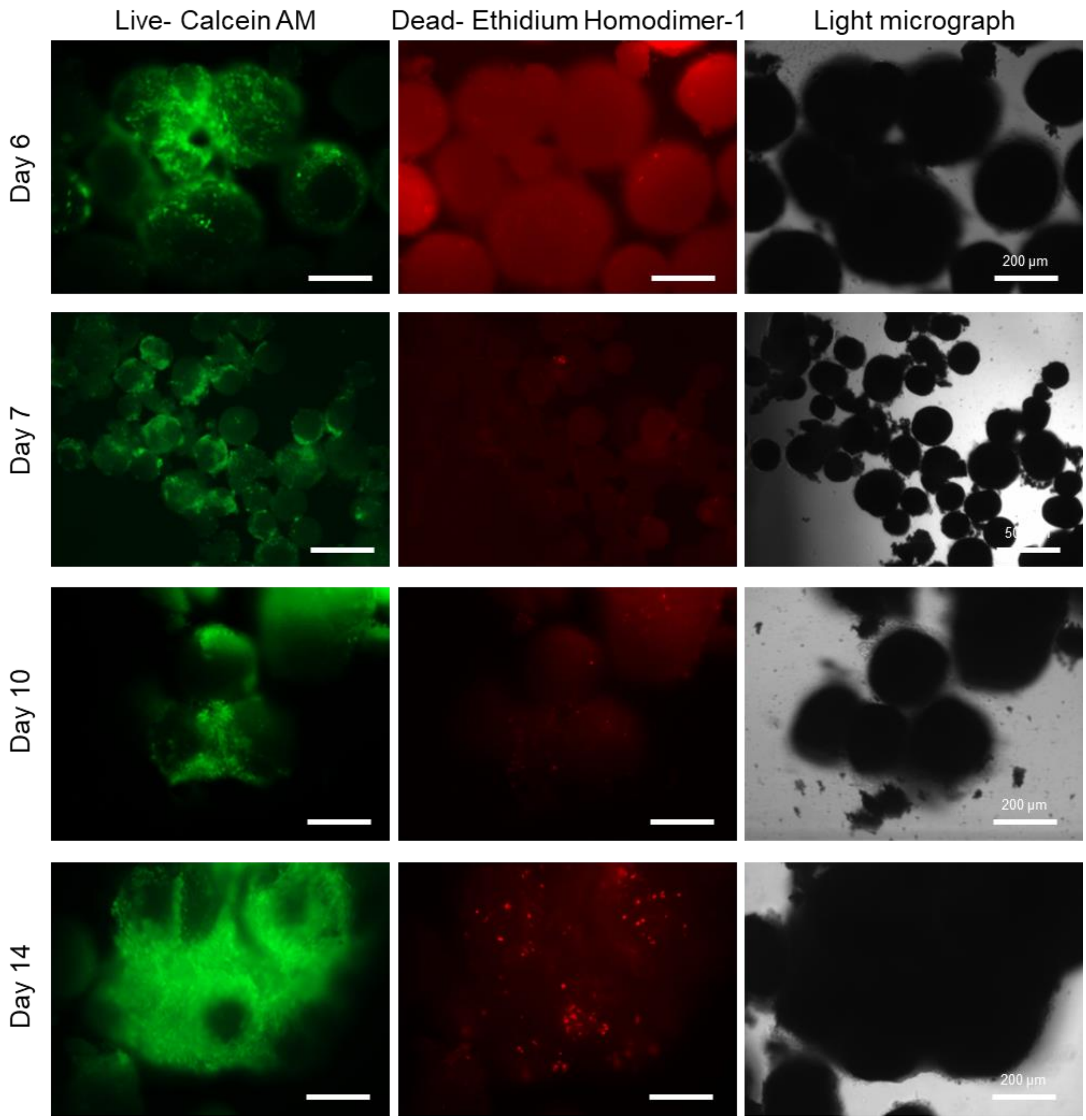

Figure S3: 14-day time course of Live/Dead stained MSC on 20% $BaSO_4$ loaded-microspheres. Images were acquired on a fluorescent microscope.

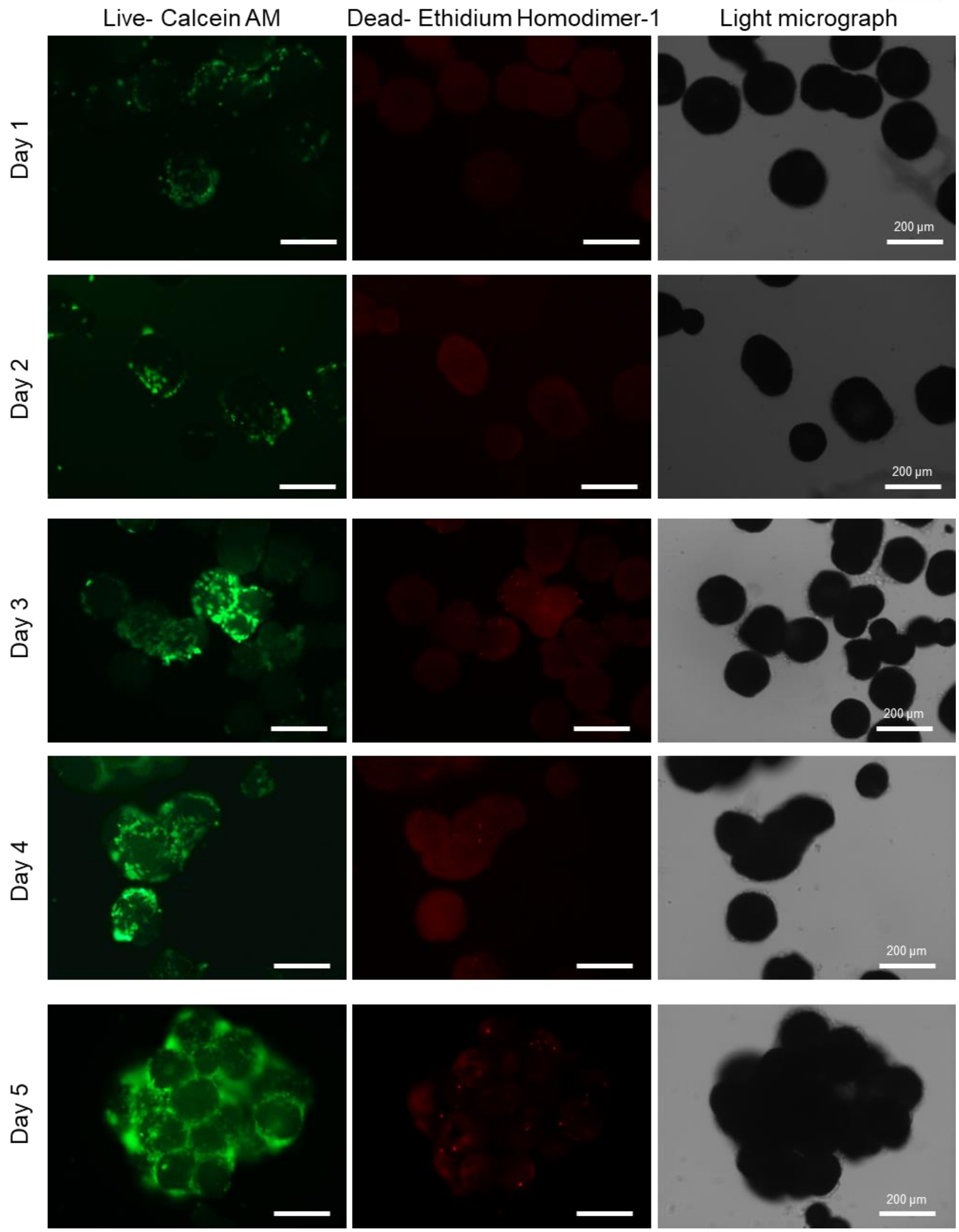
Live- Calcein AM
Dead- Ethidium Homodimer-1
Light micrograph
Day 1
Day 2
Day 3
Day 4
Day 5
200 µm

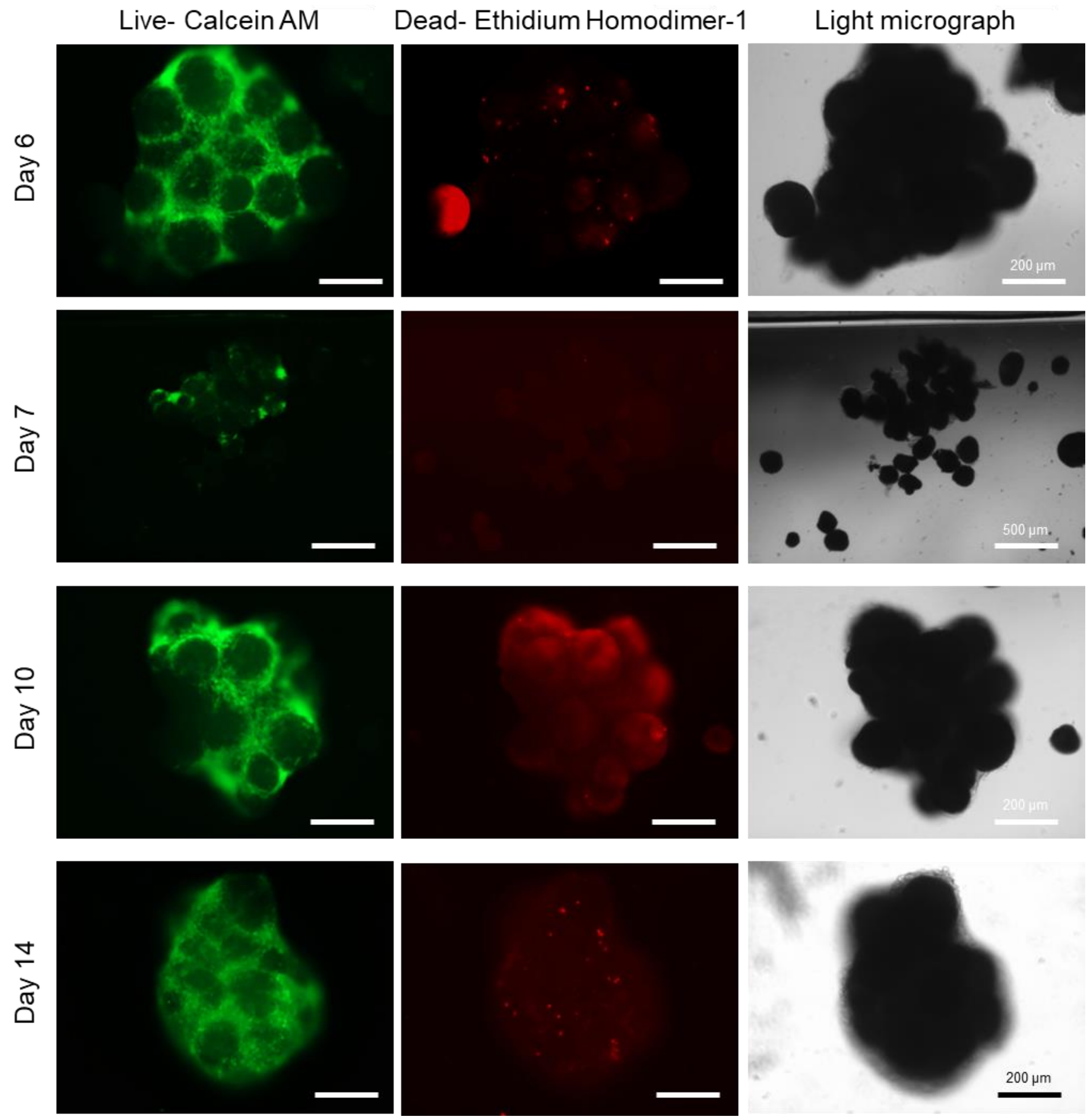

Figure S4: 14-day time course of Live/Dead stained MSC on control microspheres. Images were acquired on a fluorescent microscope.

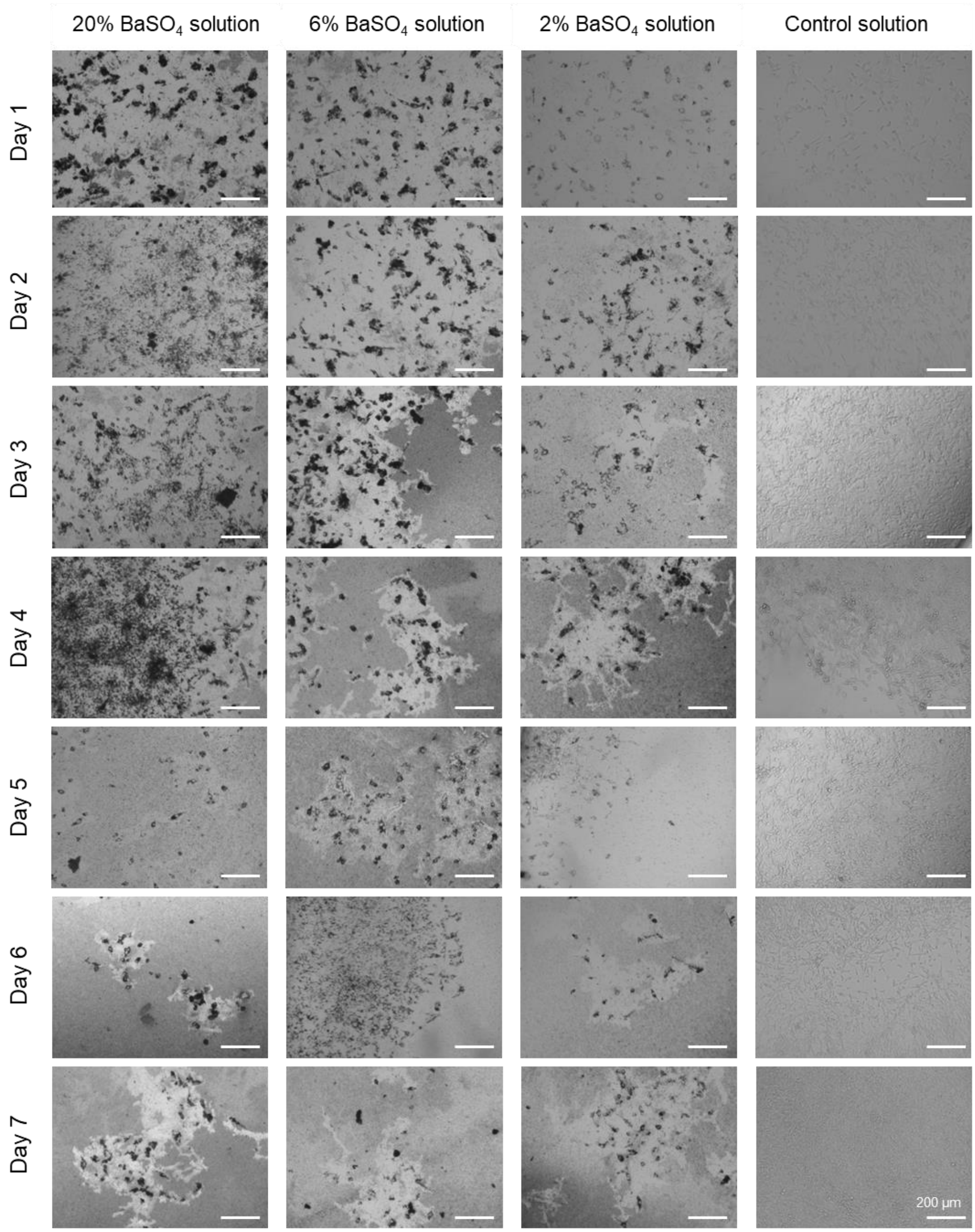
20% $BaSO_4$ solution
6% $BaSO_4$ solution
2% $BaSO_4$ solution
Control solution
Day 1
Day 2
Day 3
Day 4
Day 5
Day 6
Day 7
200 µm

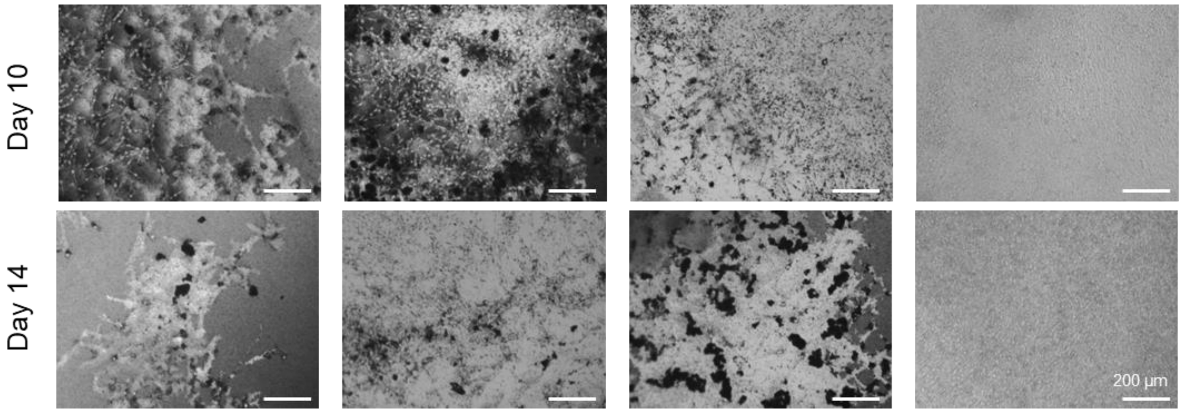

Figure S5: Cytotoxicity of $BaSO_4$ solutions to MSC culture. 2%, 6% and 20% (w/v) $BaSO_4$ solutions were added to transwell culture inserts above MSC cultures. Light micrographs were acquired over 14 days. Scalebars represent 200 µm.

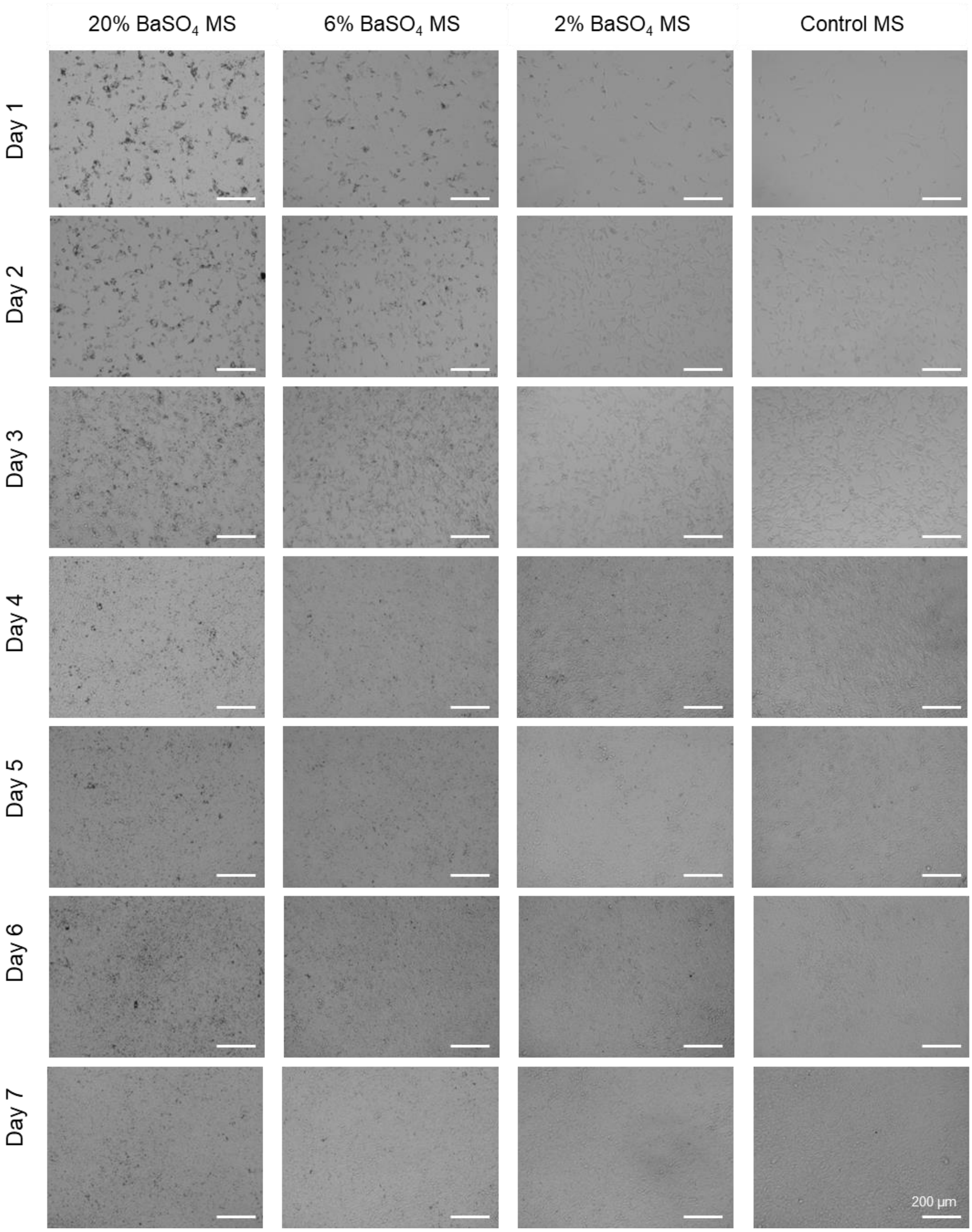
20% BaSO4 MS
6% BaSO4 MS
2% BaSO4 MS
Control MS
Day 1
Day 2
Day 3
Day 4
Day 5
Day 6
Day 7
200 µm

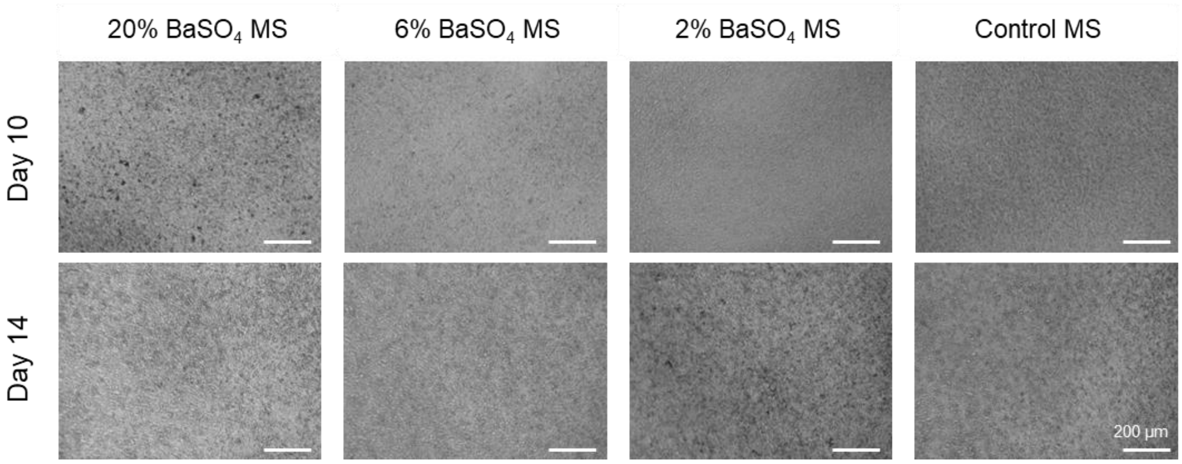

Figure S6: Cytotoxicity of $BaSO_4$ loaded microspheres to MSC culture. 2%, 6% and 20% $BaSO_4$ loaded microspheres were added to transwell culture inserts above MSC cultures. Light micrographs were acquired over 14 days. Microspheres (MS). Scalebars represent 200 µm.

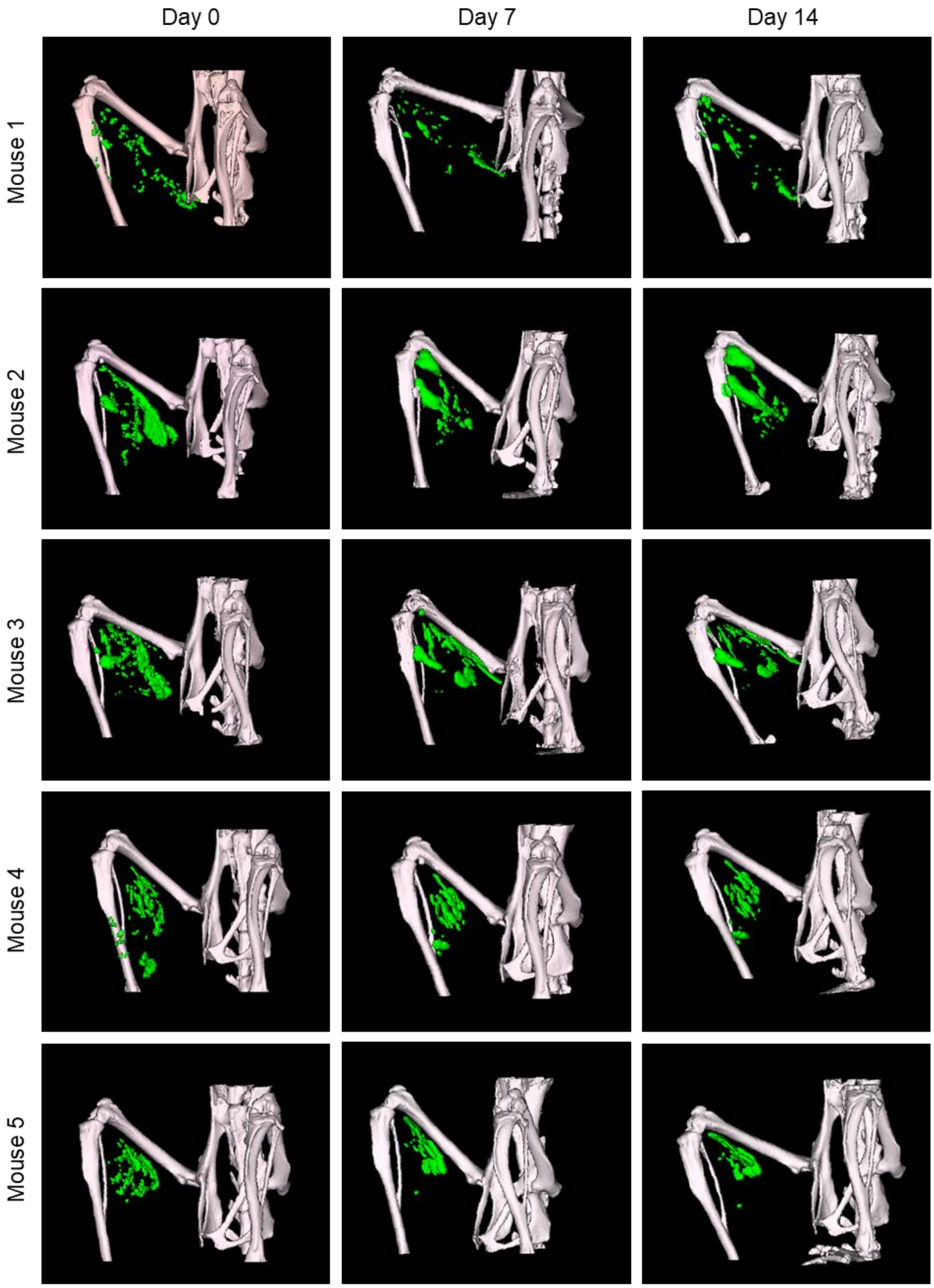

Figure S7: Reconstructed 3D volume rendered CT scans of the contrast signal emitted by $BaSO_4$ loaded microspheres in the hindlimb at day 0, 7, and 14.

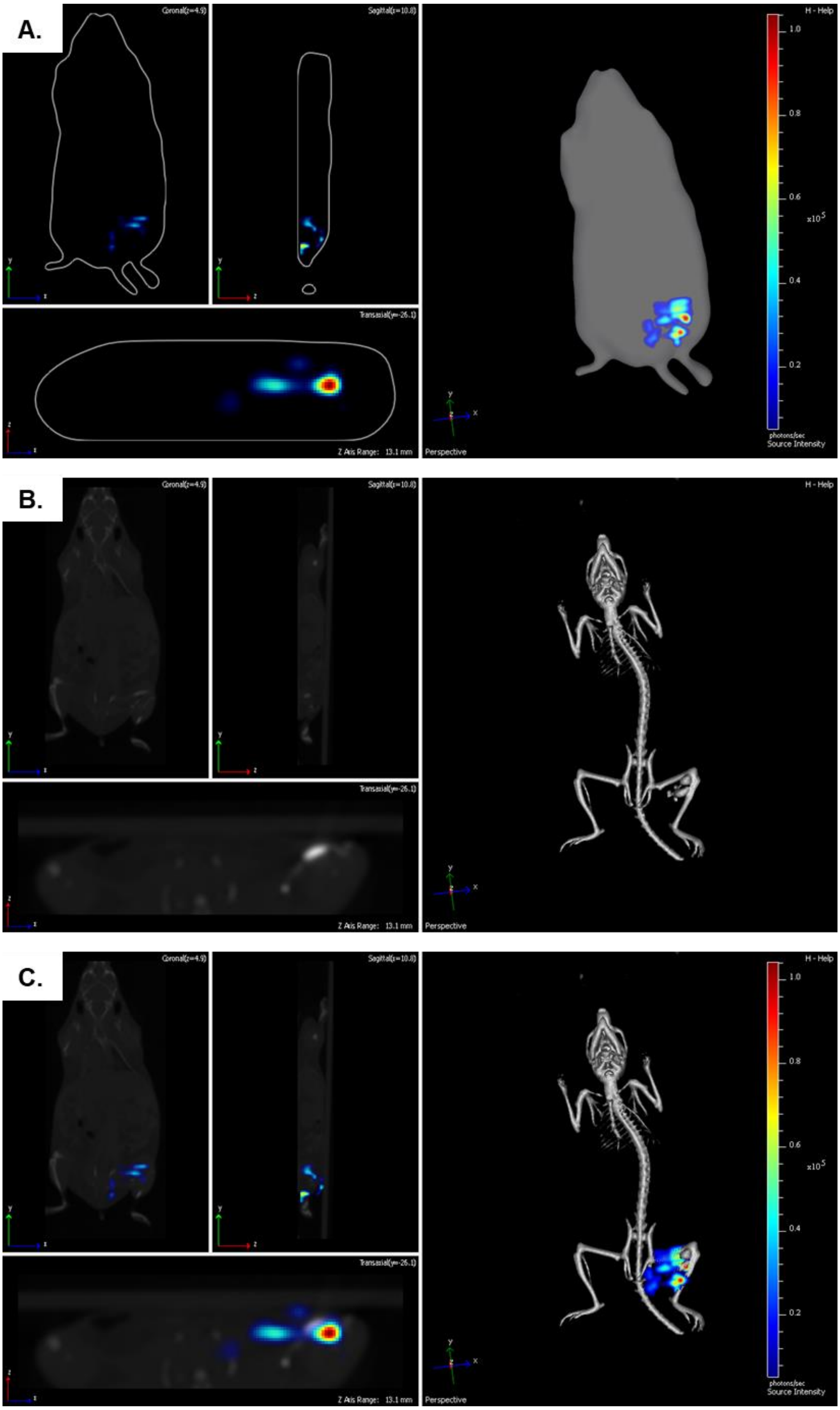

Figure S8: 2D coronal, sagittal and transaxial scans of 3D volume rendered (A) BLI, (B) μCT, and co-registered (C) reconstructions.

	1000µl resuspension			500µl resuspension		
	Pre-injection	Post-injection	6-days recovery	Pre-injection	Post-injection	6-days recovery
Ejection Fraction (%)	74.74	69.22	79.06	78.83	73.90	77.51
Fractional Shortening (%)	44.81	39.99	48.73	48.70	43.96	47.35
LV Mass (mg)	640.58	695.33	619.17	612.98	683.20	793.20
LV volume, at diastole (µl)	244.10	251.38	199.74	248.53	212.59	219.98
LV volume, at systole (µl)	71.63	66.07	56.84	48.12	58.12	58.86

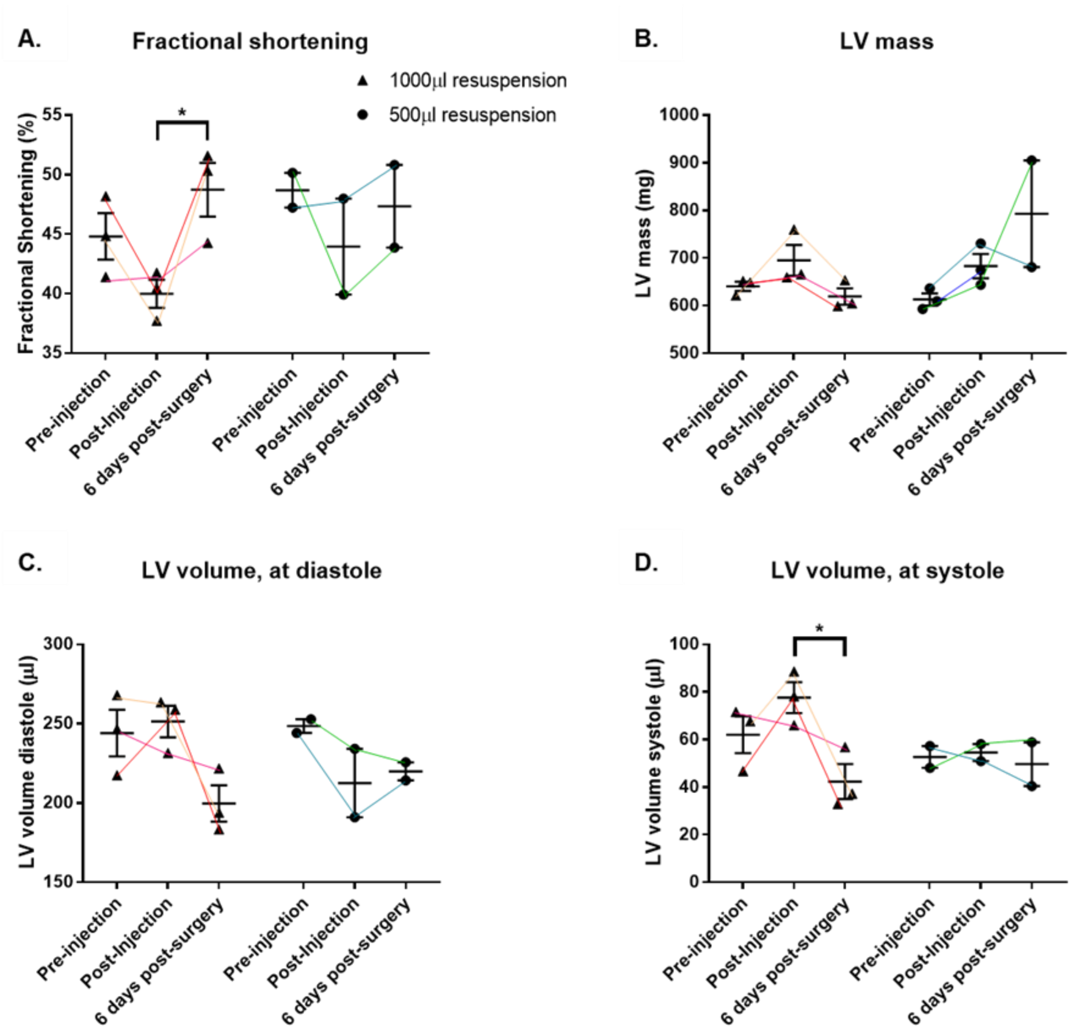

Figure S9: Summary of cardiac function analysis. Table values are the mean reported changes in (A) fractional shortening, (B) LV mass, LV volume at (C) diastole and (D) systole. Additional measures of cardiac function were measured by ultrasound analysis, before and after $BaSO_4$-loaded microspheres implantation. Data are presented as mean ±SD. The significance of the data was calculated two-way ANOVA with Tukey's post-hoc correction. (n=2-3, *P<0.05).

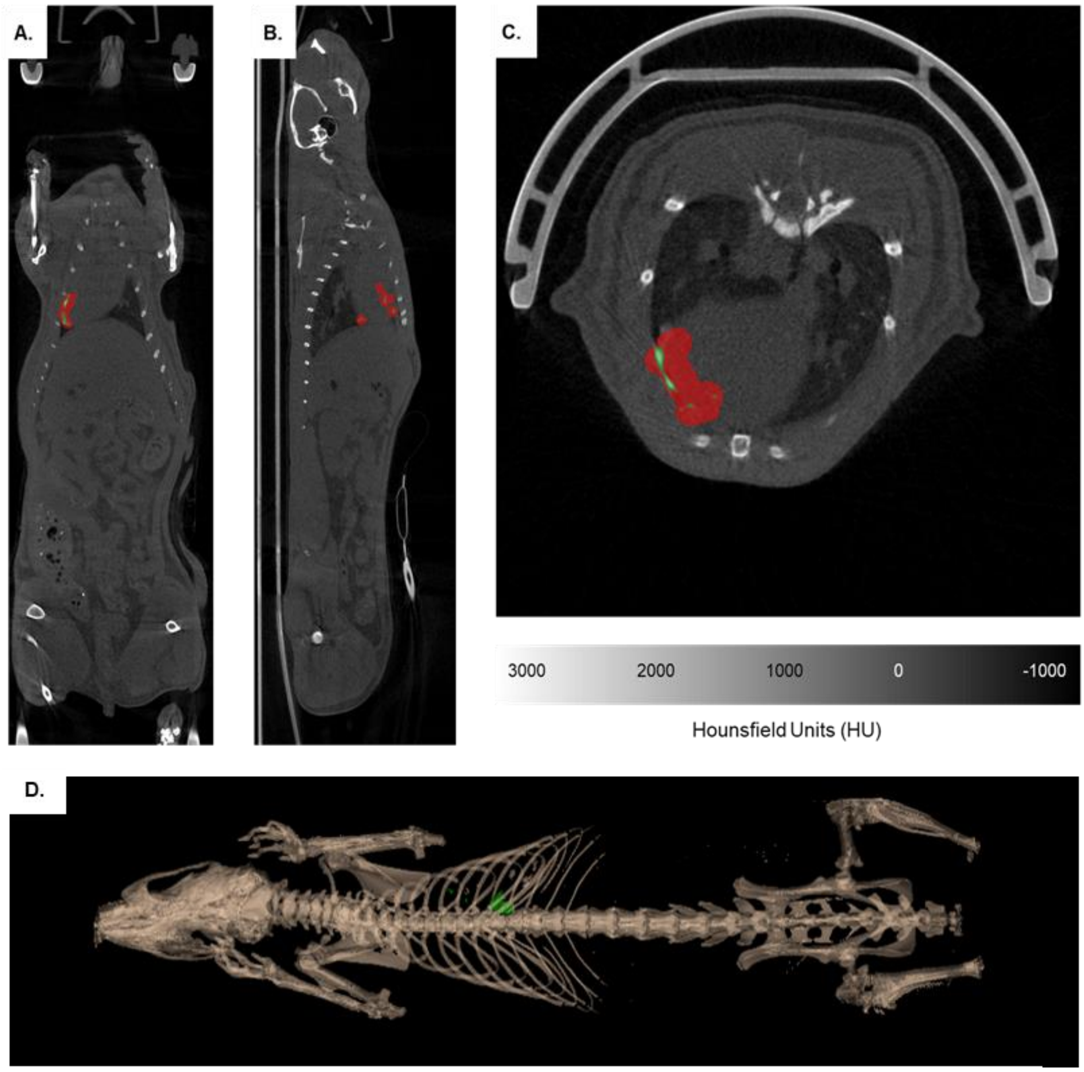

Figure S10: The signal from implanted $BaSO_4$ loaded microspheres retains contrast in the heart. Example ROI in the (A) sagittal, (B) coronal and (C) transverse cross sections used for signal analysis, and derived microspheres in green. (D) Reconstructed 3D volume rendered whole body CT scan.

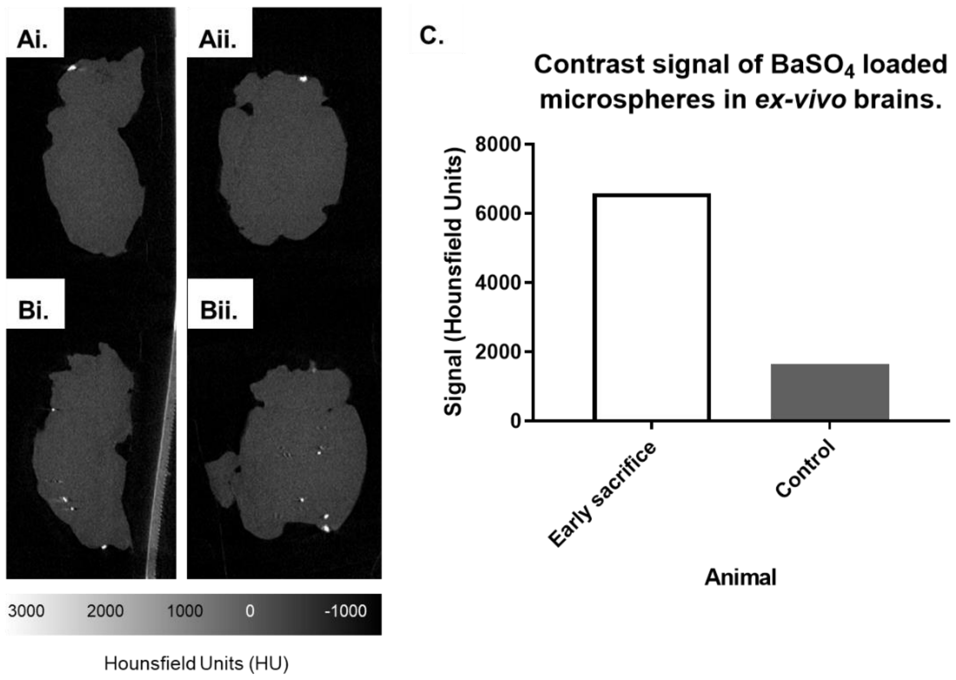

Figure S11: Detection of $BaSO_4$ generated contrast in the brain. CT cross sections of the *ex vivo* brains of (A) control and (B) sacrificed rat in (i) sagittal and (ii) coronal view, and (C) quantification of the contrast signal from respective brains. n=1.

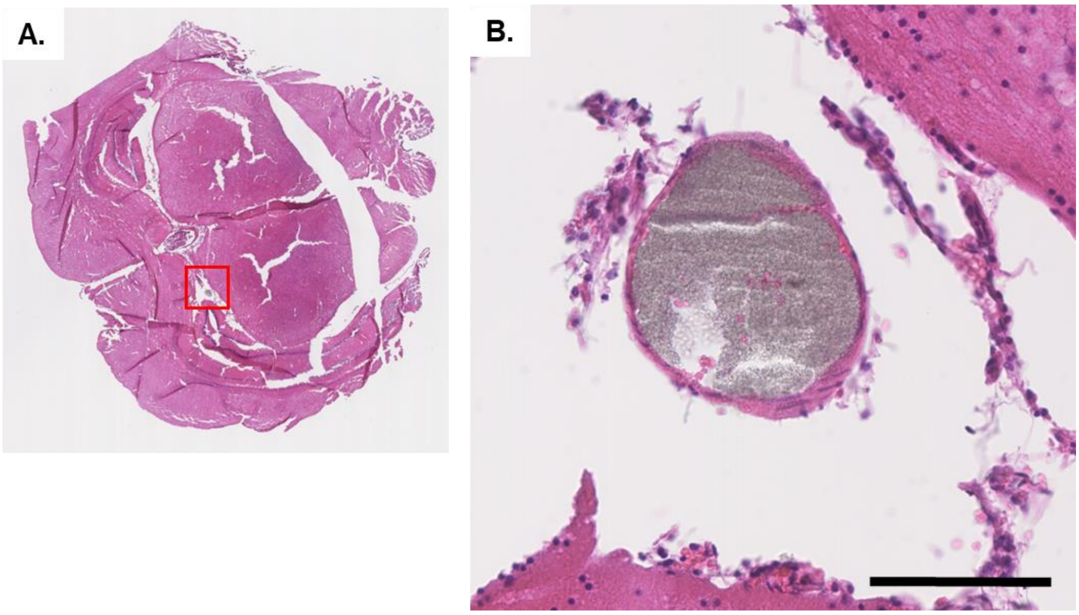

Figure S12: H&E stained section shows $BaSO_4$ loaded microspheres in a blood vessel of brain. (A) Tissue cross section shows the location of the blood vessel containing the microsphere. (B) The higher magnification image shows the microsphere within the blood vessel, with cells present on the surface and within the biomaterial. 100 µm scale bar.

A. Changes in diameter post-wetting

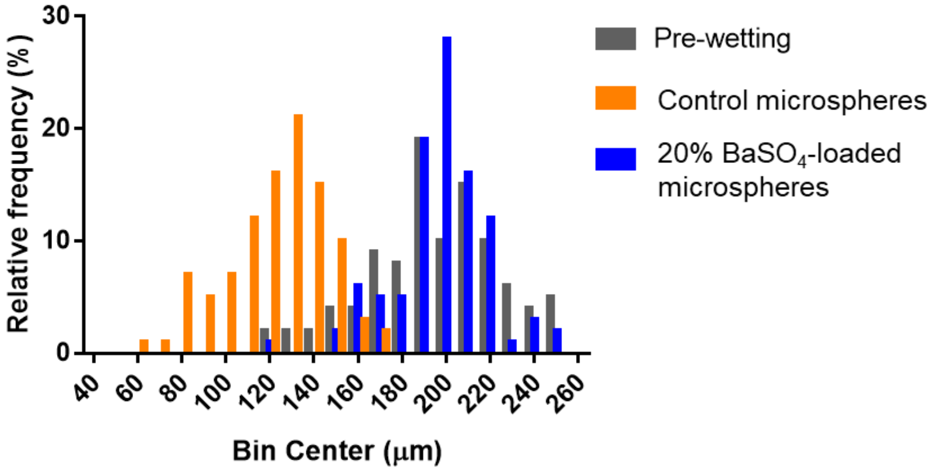

B. Changes in circularity post-wetting

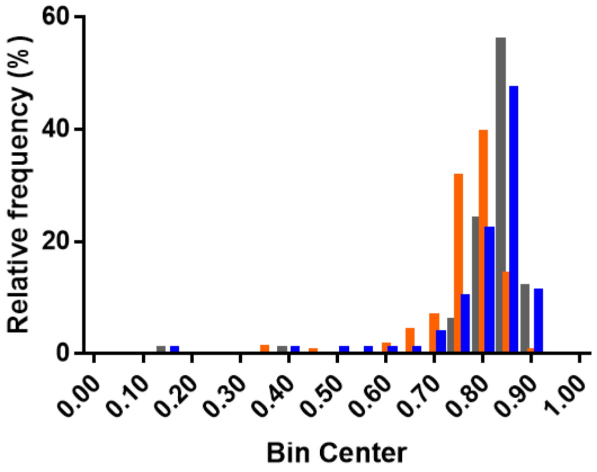

C. Calculated changes in surface area

	Pre-wetting	Control microspheres	20% $BaSO_4$-loaded microspheres
Median diameter (μm)	194.94	79.36	198.258
Calculated surface area (μm^2)	11.94×10^4	1.98×10^4	12.35×10^4

Figure S13: 20% $BaSO_4$-loaded microspheres <250 μm do not shrink at the extent as control microspheres. Changes in (A) diameter, (B) circularity, and (C) surface area after 1 day of wetting. n=100.
