# Supplementary figures and images for: CT‐Visible Microspheres Enable Whole‐Body In Vivo Tracking of Injectable Tissue Engineering Scaffolds
Source: Adv Healthc Mater. 2024 May 4;13(17):2303588. doi: 10.1002/adhm.202303588 (PMC11468734; doi:10.1002/adhm.202303588)

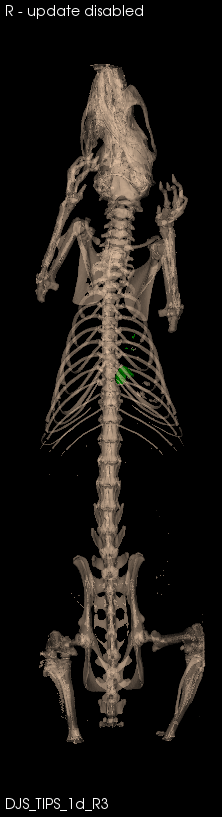

Supplement: Supplementary file 4 — Supporting Information Video 3 [file ADHM-13-2303588-s004.gif]
